# Molecular analysis of inherited disorders of cornification in polish patients show novel variants and functional data and provokes questions on the significance of secondary findings

**DOI:** 10.1186/s13023-024-03395-4

**Published:** 2024-11-05

**Authors:** Katarzyna Wertheim-Tysarowska, Katarzyna Osipowicz, Katarzyna Woźniak, Justyna Sawicka, Adrianna Mika, Anna Kutkowska-Kaźmierczak, Katarzyna Niepokój, Agnieszka Sobczyńska-Tomaszewska, Bartłomiej Wawrzycki, Aldona Pietrzak, Robert Śmigiel, Bartosz Wojtaś, Bartłomiej Gielniewski, Alicja Szabelska-Beresewicz, Joanna Zyprych-Walczak, Agnieszka Magdalena Rygiel, Alicja Domaszewicz, Natalia Braun-Walicka, Alicja Grabarczyk, Sylwia Rzońca-Niewczas, Ruszkowska Lidia, Mateusz Dawidziuk, Dominik Domański, Tomasz Gambin, Monika Jackiewicz, Katarzyna Duk, Barbara Dorożko, Orest Szczygielski, Natalia Krześniak, Bartłomiej H Noszczyk, Ewa Obersztyn, Jolanta Wierzba, Artur Barczyk, Jennifer Castaneda, Anna Eckersdorf-Mastalerz, Anna Jakubiuk-Tomaszuk, Paweł Własienko, Ilona Jaszczuk, Aleksandra Jezela-Stanek, Jakub Klapecki, Michel van Geel, Cezary Kowalewski, Jerzy Bal, Antoni Gostyński

**Affiliations:** 1 Department of Medical Genetics, Institute of Mother and Child, Warsaw, 01-211 Poland; 2 Department of Dermatology, Immunodermatology and Venereology, Medical University of Warsaw, Warsaw, 02-008 Poland; 3 Department of Pharmaceutical Biochemistry, Medical University of Gdansk, Gdansk, 80-211 Poland; 4 Medgen - MedGen Diagnostic Laboratory, MedGen Medical Center, ul. Wiktorii Wiedeńskiej 9a, Warsaw, 02- 954 Poland; 5 Department of Dermatology, Venereology, and Paediatric Dermatology, Medical University of Lublin, Staszica 11, Lublin, 20-080 Poland; 6 Department of Pediatrics, Endocrinology, Diabetology and Metabolic Diseases, Wroclaw Medical University, Wroclaw, Poland; 7 Laboratory of Sequencing, Nencki Institute of Experimental Biology, Wroclaw, Poland; 8 Laboratory of Molecular Neurobiology, Nencki Institute of Experimental Biology, Wroclaw, Poland; 9 Department of Mathematical and Statistical Methods, Poznań University of Life Sciences, Poznań, 60-637 Poland; 10 Międzyleski Specialistic Hospital In Warsaw, Bursztynowa 2, Warsaw, 04-749 Poland; 11 Department of Dermatology, Maastricht University Medical Centre +, Maastricht, The Netherlands; 12 Mass Spectrometry Laboratory, Institute of Biochemistry and Biophysics, Polish Academy of Sciences, Warsaw, Poland; 13 Clinic of Surgery of Children and Adolescents, Institute of Mother and Child, Warsaw, Poland; 14 Department of Plastic and Reconstructive Surgery, Centre of Postgraduate Medical Education, Prof. W. Orlowski Memorial Hospital, Warsaw, Poland; 15 Department of Internal and Pediatric Nursing, Institute of Nursing and Midwifery, Medical University of Gdańsk, Gdańsk, Poland; 16 Medical Center Fundacja PoMoc, ul. Dąbrowskiego 87, Łódź, 93-271 Poland; 17 Medical Genetics Unit, Mastermed Medical Center, Bialystok, Poland; 18 Department of Pediatric Neurology, Medical University of Bialystok, Bialystok, Poland; 19 Department of Cancer Genetics with Cytogenetic Laboratory, Medical University of Lublin, Lublin, Poland; 20 Department of Genetics and Clinical Immunology, National Institute of Tuberculosis and Lung Diseases, 26 Plocka str, Warsaw, 01-138 Poland; 21 National Medical Institute of the Ministry of the Interior and Administration, Warsaw, Poland

**Keywords:** Genodermatoses, MeDOC, PPK, Ichthyoses, RNAseq, Genes, Variants, Secondary findings

## Abstract

**Background:**

The Mendelian Disorders of Cornification (MeDOC) comprise a large number of disorders that present with either localised (palmoplantar keratoderma, PPK) or generalised (ichthyoses) signs. The MeDOC are highly heterogenic in terms of genetics and phenotype. Consequently, diagnostic process is challenging and before implementation of the next generation sequencing, was mostly symptomatic, not causal, which limited research on those diseases. The aim of the study was to genetically characterise a cohort of 265 Polish patients with MeDOC and to get insight into the skin lesions using transcriptome and lipid profile analyses.

**Results:**

We detected causal variants in 85% (226/265) patients. In addition to the primary gene defect, a pathogenic variant in another gene involved in MeDOC pathology was identified in 23 cases. We found 150 distinct variants in 33 genes, including 32 novel and 16 recurrent (present in > 5 alleles). In 43 alleles large rearrangements were detected, including deletions in the *STS*, *SPINK5*, *CERS3* and recurrent duplication of exons 10–14 in *TGM1*. The RNA analysis using samples collected from 18 MeDOC patients and 22 controls identified 1377 differentially expressed genes - DEG. The gene ontology analysis revealed that 114 biological processes were upregulated in the MeDOC group, including i.e. epithelial cell differentiation, lipid metabolic process; homeostasis; regulation of water loss via skin; peptide cross-linking. The DEG between *TGM1* and *ALOX12B* patients, showed that RNA profile is highly similar, though fatty acid profile in epidermal scrapings of those patients showed differences e.g. for the very long chain fatty acids (VLCFAs; FAs ≥ C20), the very long-chain monounsaturated fatty acids (VLC-MUFAs, FAs ≥ C20:1) and the n6 polyunsaturated fatty acids (n6 PUFAs).

**Conclusion:**

Our results show that NGS-based analysis is an effective MeDOC diagnostic tool. The Polish MeDOC patients are heterogenic, however recurrent variants are present. The novel variants and high number of *TGM1* and *SPINK5* copy number variations give further insight into molecular pathology of MeDOC. We show that secondary variants in MeDOC-related genes are present in a significant group of patients, which should be further investigated in the context of phenotype modifiers. Finally, we provide novel RNA and lipid data that characterise molecularly MeDOC epidermis.

**Supplementary Information:**

The online version contains supplementary material available at 10.1186/s13023-024-03395-4.

## Background

The human epidermis is a stratified epithelium composed of morphologically distinct cellular layers. In the basal layer, keratinocytes divide and subsequently initiate differentiation, which involves morphological and physiological changes. Finally, in the upper layers of the epidermis, they undergo cornification, a multistep process, during which programmed cell death is initiated and live keratinocytes are transformed into apoptotic corneocytes. The dead cells, surrounded by lipid monolayers (corneocyte lipid envelopes), are embedded in lipid-protein matrix forming a very regular structure of the epidermal barrier. On the molecular level, the cornification process involves synthesis of several structural proteins, enzymes and lipids [1]. The disturbance of keratinocyte differentiation, faulty intercellular adhesion as well as quality and quantity changes in protein/lipid content lead to homeostasis disturbance and may have clinical implications. So far, pathogenic variants in over 100 genes are known to be causative for epidermal barrier dysfunction, commonly referred to Mendelian Disorders of Cornification (MeDOC).

The MeDOC comprise a large number of non-syndromic (skin-limited) and syndromic disorders (skin and other organs affected). The disruption of cornification is manifested clinically by either localised (palmoplantar keratoderma, PPK) or generalised (Ichthyoses) hyperkeratosis in the form of scaling and keratoderma often accompanied by collodion membrane, erythema, blisters, erosions, atopy, infections, and other symptoms [2, 3].

The phenotypes of patients have many similarities, like scaling and erythema. However, they can vary significantly even within the same subtype, regardless of the underlying causative gene defects [4]. This means that a clinical diagnosis cannot be easily correlated with a specific molecular defect. Therefore, before genetic analyses became available, complex laboratory tests, such as histology of skin biopsies by electron and light microscopy, immunohistochemical staining, enzymatic activity and biochemical tests were necessary to specify the diagnosis. Consequently, the diagnosis was mostly symptomatic, not causal. This aspect had further implications on research limiting the possibility of comparative characterization and therapeutic research in patients with different causes of MeDOC.

The aim of the study was to genetically characterise a cohort of 265 Polish patients with cornification disorders. We describe novel pathogenic variants and provide unique data about incidental findings in MeDOC patients, which may influence future genotype-phenotype studies. Additionally, we present the results of transcriptome and lipid analysis in skin biopsies and scrapings, to further characterise skin lesions.

## Methods

### Patients

The group of 265 patients with clinical symptoms of MeDOC were enrolled for molecular analysis. The patients were referred by clinical geneticist and dermatologists across Poland in the years 2016–2022.

Each patient gave informed consent to participate in the study. The analysis was performed in the Department of Medical Genetics and Institute of Mother and Child (IMiD, 253 cases) and in the MedGen Medical Center (12 cases).

In the years of 2016–2020 as part of the project “Change in global gene expression versus keratin and lipid profile in rare skin disorders from the group of ichthyosis. (2014/13/D/NZ5/03304 )” the patients were asked to give consent for a skin biopsy to perform functional studies. Skin biopsies and scrapings of 18 MeDOC patients were taken (Additional file 1, part a). The 22 control skin samples were collected from persons without clinical signs of cornification disorders during surgical procedures performed for other medical indications. In the case of 10 patients with *ALOX12B* (*n* = 5) and *TGM1* (*n* = 5), from whom the biopsies were taken, we obtained detailed clinical questionnaire (Additional file 1, part b). These patients manifest typical features of ARCI (autosomal recessive congenital ichthyosis), although pain, keratosis pilaris, nail changes and sparse hair were more prevalent in *TGM1* patients.

### Genotyping

#### The Next Generation sequencing (NGS) panel

In the majority of patients (245 cases) genotyping was performed using a customized NGS panel covering all coding exons of 60 genes: *AAGAB*,* ABCA12*,* ABHD5*,* ADAM10*,* ALDH3A2*,* ALOX12B*,* ALOXE3*,* AP1S1*,* AQP5*,* CDSN*,* CLDN1*,* CSTA*,* CTSC*,* CYP4F22*,* DSG1*,* DSP*,* EBP*,* ENPP1*,* ERCC2*,* ERCC3*,* FERMT1*,* FLG*, *GJA1*,* GJB2*,* GJB3*,* GJB4*,* GTF2H5*,* HOXC13*,* JUP*,* KANK2*,* KRT1*,* KRT10*,* KRT16*,* KRT17*,* KRT2*,* KRT9*,* LIPN*,* LOR*,* MBTPS2*,* MPLKIP*,* NIPAL4*,* NSDHL*,* PEX7*,* PHYH*,* PKP1*,* PNPLA1*,* POFUT1*,* POGLUT1*,* POMP*,* SERPINB7*,* SLC27A4*,* SLURP1*,* SNAP29*,* SPINK5*,* ST14*,* STS*,* SUMF1*,* TGM1*,* TRPV3*,* VPS33B*. The panels were supplied by Roche and Agilent. The libraries were made using the KAPA Library Preparation Kit and SureSelect Kit. The MiSeq sequencer (Illumina) was used to sequence the samples. The bioinformatic analysis was performed using pipeline based on Ensembl Variant Effect Predictor (VEP). The reads were aligned against the GRCh38 human genome assembly. The following databases were used for variant annotations: the SNPdb (NCBI), Ensembl, OMIM, GnomAD, ClinVar, and HGMD Professional. The Copy Number Variation (CNV) analysis was performed using CODEX, CoNIFER, cn.mops, ExomeDepth and XHMM algorithms.

The clinical significance of the variants was assessed according to ACMG [5] using Varsome (https://varsome.com/) and Franklin (https://franklin.genoox.com) online software packages.

## Verification of NGS results

### All variants detected by NGS were verified as described below

Whenever possible, biparental origin of the variants was verified by testing the parents (*n* = 122 families, data not shown).

#### Single nucleotide variants: Sanger sequencing

The presence of all pathogenic and likely pathogenic variants detected using NGS analysis was confirmed by Sanger sequencing. The primers were designed using primer3 software (https://primer3.ut.ee/) and are available upon requests together with PCR conditions.

Rare *FLG* (filaggrin) variants confirmation (apart from p.Arg501Ter and p.Ser761CysfsTer36) was performed in the Departments of Dermatology and Clinical Genetics at the Maastricht University Medical Centre + as described before [6].

#### Copy number variation

The *TGM1* duplication was confirmed by qPCR using TaqMan Copy Number Assay (ThermoFisher Scientific). The probes Hs01865876_cn (intron 12-exon 13 boundary, Chr.14:24249114–24263210 on Build GRCh38) and Hs03052754_cn (exon 14, Chr.14:24249114–24263210 on Build GRCh38) were used to analyse the *TGM1* gene, the control probe was TaqMan™ Copy Number Reference Assay, human, *TERT* (ThermoFisher Scientific). Reactions were performed according to the manufacturer’s recommendations using the CFX Opus 96 Real-Time PCR System (BioRad).

The presence of deletions was confirmed by analysis of the parental samples using the NGS gene panel or, in case of *STS*, by Multiplex Ligation-dependent Probe Amplification (MLPA), see below.

### MLPA analysis of the *STS* gene

The *STS* gene analysis was performed using MLPA in 9 patients with the suspicion of X-linked ichthyosis. It was also used to confirm deletions that were detected by NGS. The MLPA was performed using The SALSA MLPA Probemix P160 STS (MRC Holland) on Sequencer ABI 3500. The results were analysed using Coffalyser.Net™ (MRC Holland).

### Analysis of the most common variants in the *FLG* gene

In 11 cases, analysis of the two most common pathogenic variants p.Arg501Ter and p.Ser761CysfsTer36 in *FLG* was performed by Sanger sequencing using primers and PCR conditions as described by Sandilands et al. [7].

## Functional studies

### Skin biopsy and scrapings

The 3 mm skin biopsies were taken from the patients and controls for RNA sequencing, while for lipid analysis, only the scrapings were collected mechanically using a scalpel. The biopsies and scrapings were snap frozen immediately. The epidermis was detached mechanically in a cryotome prior to further studies. All samples were stored at -80 C.

### RNA sequencing

The RNA isolation and sequencing were performed as we described previously [8]. Briefly, RNA was isolated from homogenized epidermis with RNeasy Micro Kit (Qiagen) and quantified on Agilent 2100 Bioanalyzer using an RNA 6000 Pico Kit (Agilent Technologies, Ltd.). The polyA enriched RNA libraries were prepared using the QuantSeq 3’ mRNA-Seq Library Prep Kit according to the manufacturer’s protocol (Lexogen GmbH, Vienna, Austria) and the libraries were single-end sequenced (75 bp) on a HiSeq 1500 (Illumina, San Diego, CA 92122 USA). Reads were aligned to the reference human genome GRCh38 (Ensembl database) using STAR aligner and counted using HT seq. Genes with exceptionally low expression (less than 5 reads across all samples) were excluded from further analysis. Linear models implemented in the edgeR package were used for normalization and differential expression analysis. The adjusted p-value (FDR) < 0.05 and |logFC| > 2 were used to determine the list of differentially expressed genes. An enrichment test implemented in the systemPipeR R package was used for gene ontology (GO) enrichment analysis. The genes that were considered in this type of analysis were those with |logFC| > 1.5 and an adjusted p-value (FDR) < 0.05. The returned corresponding Bonferroni adjusted p-value < 0.05 was used as the significance threshold for each GO. The R version 3.6.2 was used for all statistical analysis. The genes considered for GO analysis, were also included in Reactome’s Pathway Analysis based on hypergeometric distribution for pathway over-representation analysis. The Reactome version 87 on 27/12/2023 was used.

### Fatty acid (FA) analysis

Total lipids were extracted according to the Folch method [9]. Subsequently, the lipid extracts were dried under a stream of nitrogen and hydrolyzed with 0.5 M KOH at 90 °C for 3 h. The solution was acidified and 1mL of water was added. The unesterified fatty acids (FAs) were extracted with n-hexane (3 × 1 mL) and the solvent was then completely evaporated under a stream of nitrogen. To obtain fatty acid methyl esters (FAMEs), a 10% boron trifluoride–methanol solution was added to each sample and heated at 55 °C for 90 min. Then, 1mL of water was added to the reaction mixture and FA derivatives were extracted with n‐hexane (3 × 1 mL). 19 methyleicosanoic acid was used as an internal standard. The analysis of FAs was performed by gas chromatography – mass spectrometry (GC-MS) [10].

## Results

## Genotyping

We detected causal variants for clinical phenotypes in 226/265 patients, which gave an overall diagnostic yield of 85%. The different detection rates were achieved, with respect to the method used (Table 1).

**Table 1:** Results of genotyping performed using different diagnostic strategies

	Total number of patients	Number of patients with causative pathogenic variants identified	Number of patients with one pathogenic variant in AR inheritance or no variants at all (%)
TOTAL:	265	226 (85%)	39 (15%)
NGS customized panel:	245	214 (87%)	31 (13%)
*STS* analysis by MLPA	9	8 (89%)	1 (11%)
*FLG* - two mutations test by Sanger	11	4 (36%)	7 (64%)

In 214/245 (87%) patients analysed by NGS, the genetic cause of the disease was elucidated (Table 1 and Additional file 2). In 31/245 (13%) cases either one pathogenic/likely pathogenic variant was found (while autosomal recessive (AR) inheritance was expected) or no pathogenic variants were identified. In 9 cases, only the *STS* gene analysis by MLPA was performed enabling for detection of *STS* gene deletions in 8/9 (89%) cases. In a subgroup of 11 patients, we only performed limited genetic analysis comprising the two most common *FLG* variants: p.Arg501Ter and p.Ser761CysfsTer36. Two pathogenic variants were detected in 4/11 (36%) cases.

In 23 cases of 245 analysed by NGS, in addition to the primary gene defect, a pathogenic variant(s) in another gene(s) were detected (Table 2). The majority of secondary findings (17/23) were variants already reported in the ClinVar database as pathogenic (P) or likely pathogenic (LP). One variant has the conflicting status (LP vs. variants of unknown significance, VUS) and 3 are reported as VUS. In 3 cases novel variants were found, while in one patient (No #19 in Table 2), known pathogenic variants were found in two other genes.

**Table 2:** The primary genotypes and secondary findings

No (sex)	Gene and variants found causative of phenotype			Other (*P*, LP and VUS) findings in NGS panel (all variants were detected as heterozygotes)		
	gene	allele 1	allele 2	gene	allele	Classification in ClinVar
1	*ALOX12B*	c.1454T>C p.(Phe485Ser)	c.1562A>G p.(Tyr521Cys)	*TGM1*	c.377G>A p.(Arg126His)	known pathogenic VCV000279909.12
2	*ALOXE3*	c.700 C>T p.(Arg234Ter)	c.700 C>T p.(Arg234Ter)	*ERCC3*	c.460C>T p.(Gln154Ter)	known pathogenic VCV000801749.3
3	*ALOX12B*	c.1562A>G p.(Tyr521Cys)	c.1562A>G p.(Tyr521Cys)	*ERCC3*	c.325C>T p.(Arg109Ter)	known pathogenic VCV000265515.32
4	*ABCA12*	c.4139A>G p.(Asn1380Ser)	c.6100A>G p.(Asn2034Asp)	*FLG*	c.7339C>T p.(Arg2447Ter)	pathogenic/likely pathogenic VCV000050932.46
5	*ALOX12B*	c.1265C>T p.(Pro422Leu)	c.1562A>G p.(Tyr521Cys)	*FLG*	c.2282_2285del p.(Ser761CysfsTer36)	known pathogenic VCV000016320.59
6	*ALOX12B*	c.1562A>G p.(Tyr521Cys)	c.2094C>A p.(Ser698Arg)	*FLG*	c.2282_2285del p.(Ser761CysfsTer36)	known pathogenic VCV000016320.59
7	*ALOXE3*	c.700C>T p.(Arg234Ter)	c.1472T>G p.(Leu491Arg)	*FLG*	c.5702del p.(Gly1901AlafsTer194)	NOVEL*
8	*TGM1*	c.1135G>C p.(Val379Leu)	c.(1402 + 1_1401-1)_(2225 + 1_2226-1)dup p.?	*FLG*	c.2282_2285del p.(Ser761CysfsTer36)	known pathogenic VCV000016320.59
9	*TGM1*	c.1135G>C p.(Val379Leu)	c.1500T>A p.(Ser500Arg)	*FLG*	c.3982C >T p.(Gln1328Ter)	NOVEL*
10	*ALOX12B*	c.1A>G p.(Met1?)	c.2094C>A p.(Ser698Arg)	*FLG*	c.2282_2285del p.(Ser761CysfsTer36)	known pathogenic VCV000016320.59
11	*STS*	c.(?_-1)_(*1_? )del p.1Met_583Terdel	(-)	*FLG*	c.2282_2285del p.(Ser761CysfsTer36)	known pathogenic VCV000016320.59
12	*KRT10*	c.467G>A p.(Arg156His)	(-)	*FLG*	c.2282_2285del p.(Ser761CysfsTer36)	known pathogenic VCV000016320.59
13	*KRT2*	c.1459G>A p.(Glu487Lys)	(-)	*ABCA12*	c.6611G>A p.(Arg2204Gln)	VUS RCV001136974.4
14	*STS*	c.(?_-1)_(*1_? )del p.1Met_583Terdel		*ABCA12*	c.179G>C p.(Arg60Pro)	VUS/likely pathogenic VCV000264998.5
15	*SPINK5*	c.(?_-1)_(410 + 1_411-1)del p.?	c.(?_-1)_(410 + 1_411-1)del p.?	*ALOXE3*	c.1432 A>C p.(Ser478Arg)	VUS RCV001127791.4
16	*KRT2*	c.566T>C p.(Phe189Ser)	(-)	*ALOX12B*	c.1562 A>G p.(Tyr521Cys)	pathogenic VCV000039546.40
17	*ALOXE3*	c.700C>T p.(Arg234Ter)	c.1889C>T p.(Pro630Leu)	*VPS33B*	c.199T>C p.(Tyr67His)	NOVEL*
18	*SPINK5*	c.1825C>T p.(Gln609Ter)	c.(?_-1)_(1479 + 1_1480-1)del p.?	*GTF2H5*	c.49 A>T p.(Lys17Ter)	pathogenic VCV000975159.1
19	*SLC27A4*	c.1541A>G p.(Glu514Gly)	c.1510C>T p.(Arg504Cys)	*FLG*	c.7339C>T p.(Arg2447Ter)	pathogenic/likely pathogenic VCV000050932.46
				*NIPAL4*	c.341C>A p.(Ala114Asp)	pathogenic/likely pathogenic VCV000001731.29
20	*ALOXE3*	c.700C>T p.(Arg234Ter)	c.700C>T p.(Arg234Ter)	*DSP*	c.4198C>T p.(Arg1400Ter)	pathogenic/likely pathogenic VCV000199884.19
21	*ALOX12B*	c.467_470dup p.(His158CysfsTer20)	c.1562A>G p.(Tyr521Cys)	*TGM1*	c.1631A>G p.(Tyr544Cys)	VUS RCV000664924.1
22	*ALOX12B*	c.1207C>T p.(His403Tyr)	c.1790C>A p.(Ala597Glu)	*WNT10A*	c.321C >A p.(Cys107Ter)	pathogenic VCV000004461.41
23 (F)	*FLG*	c.1501C>T p.(Arg501Ter)	c.7339C>T p.(Arg2447Ter)	*STS*	c.1316A>G p.(His439Arg)	pathogenic VCV000010556.1

* see Table 3 ACMG classification

### Variant characteristics

In total, we detected 150 distinct variants in 415 alleles of 33 genes (Table 2, Additional file 2 and 3). The alleles with pathogenic/likely pathogenic variants in the *ALOX12B* gene were the mostly represented (*n* = 115), followed by variants in the *TGM1* (*n* = 68), *FLG* (*n* = 39), *ALOXE3* (*n* = 36), and *STS* (*n* = 27) genes. The number of distinct variants was also the highest in the *TGM1* (*n* = 23) and the *ALOX12B* genes (*n* = 22). Among 150 distinct variants detected, 32 were previously undescribed and 118 known. The most common was c.1562 A > G p.(Tyr521Cys) in the *ALOX12B* gene, detected in 52 alleles. Eight variants were detected in over 10 alleles, and total of 51 were detected at least 2 times (Additional file 4). Importantly, 99 variants (66%) were unique. Among 32 novel ones, as many as 9 were found in *ALOX12B*, followed by 4 in *ALOXE3*, 4 in *KRT1*, and 3 in *ABCA12* and *KRT10* (Table 3).

**Table 3:** The variants identified for the first time in this study

GENE (numer of variants)	VARIANT	Number of alleles with the variant	ACMG classification	ACMG main criteria
*ABCA12* (*n* = 3)	c.6100 A>G p.(Asn2034Asp)	1	VUS	PM2, PP3
	c.6194del p.(Asn2065ThrfsTer3)	1	likely pathogenic	PVS1, PM2
	c.758delT p.(Phe253SerfsTer27)	1	likely pathogenic	PVS1, PM2
*ALOX12B* (*n* = 9)	c.1454T>C p.(Phe485Ser)	2	VUS	PM2, PM1, PP2, PP3
	c.962T>A p.(Met321Lys)	2	VUS	PM2, PM1, PP2
	c.808 A>C p.(Asn270His)	1	VUS	PM2, PM1, PP2, PP3
	c.1154T>C p.(Val385Ala)	1	likely pathogenic	PM1, PP2, PM2, PP3
	c.1446dupC p.(Asn483GlnfsTer2)	1	likely pathogenic	PVS1, PM2
	c.1529 A>G p.(Glu510Gly)	1	VUS	PM2, PP2
	c.808 A>C p.(Asn270His)	1	VUS	PM2, PM1, PP2, PP3
	c.911T>C p.(Leu304Ser)	1	VUS	PM2, PM1, PP2, PP3
	c.1102–2 A>T p.?	1	likely pathogenic	PVS1, PM2
*ALOXE3* (*n* = 4)	c.707del p.(Leu236ArgfsTer44)	1	likely pathogenic	PVS1, PM2
	c.1472T>G p.(Leu491Arg)	1	VUS	PM2, PP3
	c.842delG p.(Gly281ValfsTer38)	1	likely pathogenic	PVS1, PM2
	c.984 C>A p.(Tyr328Ter)	1	likely pathogenic	PVS1, PM2
*CERS3* (*n* = 1)	c.(999+1_1000-10)_(*11_? )del p.?	2	VUS	Uncertain (0.75)
*DSG1* (*n* = 1)	c.518–2 A>G p.?*	1	likely pathogenic	PVS1, PM2
*FLG* (*n* = 1, SF)	c.5702del p.(Gly1901AlafsTer194)	1	likely pathogenic	PVS1, PM2
*KRT1* (*n* = 4)	c.1430T>C p.(Leu477Pro)	1	VUS	PM2, PM1
	c.1535delT p.(Ile512ThrfsTer102)*	1	likely pathogenic	PVS1, PM2
	c.539 A>T p.(Glu180Val)	1	VUS	PM2, PM1, PP3
	c.551T>A p.(Ile184Asn)	1	VUS	PM2, PM1, PP3
*KRT10* (*n* = 3)	c.1441_1448del p.(Gly481ArgfsTer97)	1	likely pathogenic	PVS1, PM2
	c.1554dupC p.(Ser519GlnfsTer62)*	1	likely pathogenic	PVS1, PM2
	c.1689_1690del p.(Ser563ArgfsX17)	1	likely pathogenic	PVS1, PM2
*SLC27A4* (*n* = 1)	c.1541 A>G p.(Glu514Gly)	1	VUS	PM2, PP3
*SPINK5* (*n* = 1)	c.(?_-1)_(410+1_411-1)del p.?	3	VUS	Uncertain (0.75)
*ST14* (*n* = 1)**	c.2086 C>A p.(Arg696Ser)**	1	VUS	PM2
*TGM1 *(*n* = 2)	c.1500T>A p.(Ser500Arg)	2	VUS	PM2
	c.1490 A>T p.(Glu497Val)	1	likely pathogenic	PP3, PM2
*VPS33B* (*n* = 1, SF)	c.199T>C p.(Tyr67His)	1	VUS	PM2, PP3

SF- variants were found as secondary findings; *phenotype details of this patient were partially published by us recently [11]; ** the variant in *ST14* gene was identified in one allele only, while defects in *ST14* cause autosomal recessive ichthyosis. Therefore molecular cause of MeDOC in this case is not known

Among all variants we found, the majority were SNV (single nucleotide variants), however, 43/415 (10.3%) alleles harboured large rearrangements (CNV – copy number variations) (Table 4). Deletion of the *STS* gene was the most common. Moreover, in 6 alleles of *SPINK5*, large deletions were found encompassing exons 1–16: c.(?_-1)_(1479 + 1_1480-1)del (in 3 alleles) and exons 1–5: (c.(?_-1)_(410 + 1_411-1)del (in 3 alleles, variant not reported before). Finally, one patient had a homozygous deletion of exon 12 in *CERS3* gene. In nine *TGM1* alleles we detected duplication of exons 10–14 (c.(1402 + 1_1401-1)_(2225 + 1_2226-1)dup).

**Table 4:** The list of CNV identified in the study

Gene	CNV	Number of alleles	Numer of patients
*STS*	c.(?_-1)_(*1_? )del p.01Met_583Terdel	26	26
*TGM1*	c.(1402 + 1_1401-1)_(2225 + 1_2226-1)dup p.?	9	9
*SPINK5*	c.(?_-1)_(1479 + 1_1480-1)del p.?	3	3
*SPINK5*	c.(?_-1)_(410 + 1_411-1)del p.?	3	2
*CERS3*	c.(999 + 1_1000-10)_(*11_? )del p.?	2	1

### Functional studies

The RNA samples were collected from 18 patients and from 22 anonymous controls without signs of a cornification disorder. There were 7 cases with pathogenic variants in *TGM1*, 5 in *ALOX12B*, 2 in *ALOXE3* and one in *LOR*,* SPINK5*,* STS* and *ABCA12*, each. Median age of the patients was: 21.5 ± 13.5. The control group did not manifest any clinical symptoms of a cornification disorder and included: 13 samples obtained from female adults (BMI 25–33, median age: 50,5 ± 20.87), 8 samples from children (BMI = 18.5–25, median age: 6.5 ± 3.5) and 1 from adult men. The characteristics of patients and controls, including skin samples collection sites and type are given in the Additional file 1.

### RNA analysis

Principal component analysis (PCA) was performed on RNA-seq expression data to verify if samples are grouping according to considered conditions (Additional file 5). The PCA revealed that MeDOC samples fall apart from healthy ones. PCA also revealed that grouping according to the skin sample collection site was not exact. In order to get insight into the expression pattern differences between epidermis of thorax and limb of the adult controls, we performed the Differentially Expressed Genes (DEG) analysis and discovered only 39 of them. We also performed DEG analysis between children and adult controls, which reached 137. Those numbers were however much smaller than the values of DEGs between patient and controls groups, which justified further analyses.

#### MeDOC patients vs. controls

**Fig. 1:**
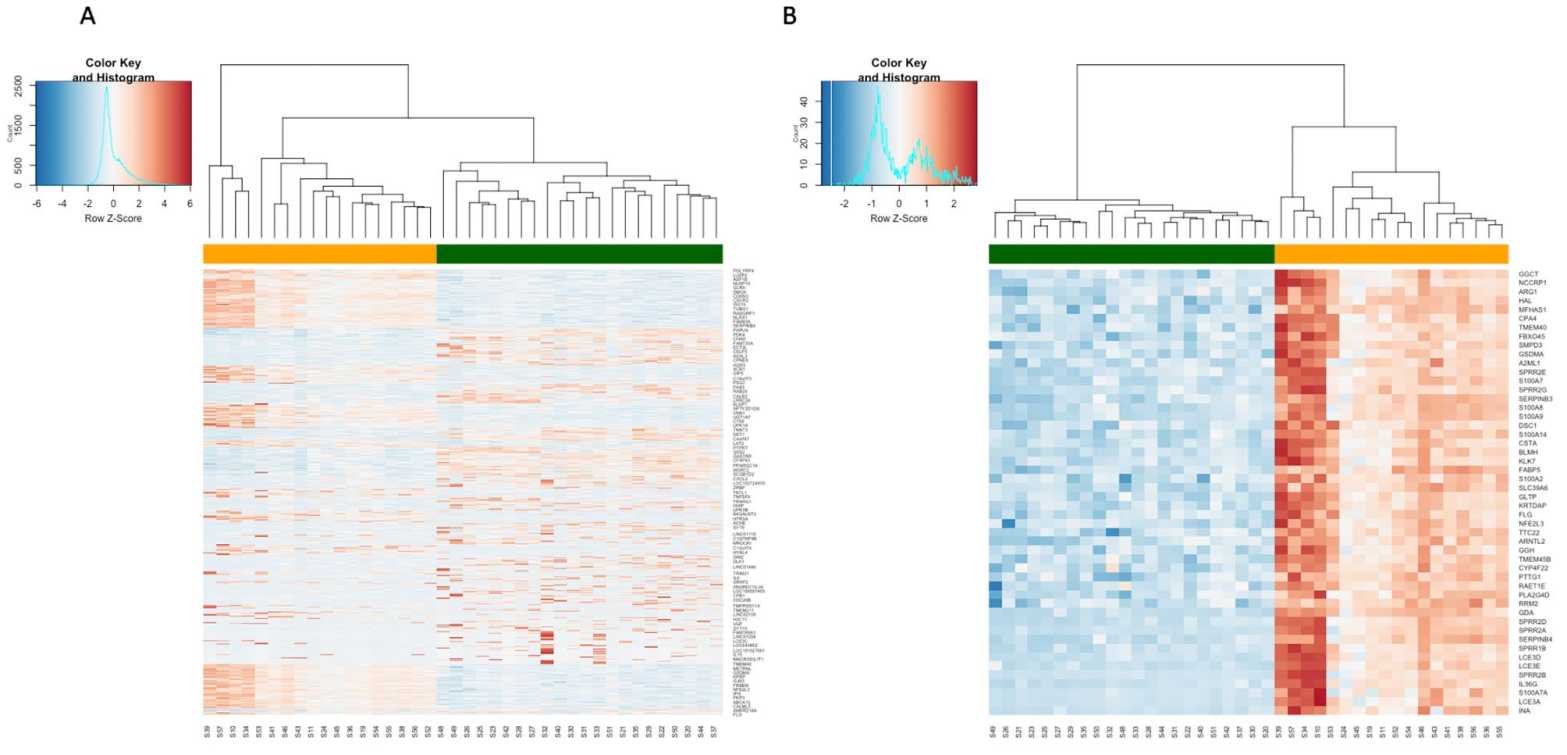
Heatmaps of DEGs patients vs. control

First, we performed the general analysis comprising all patients and control samples and defined 1377 DEG of which 647 were upregulated and 730 downregulated (Fig. 1). The statistical overrepresentation test using Reactome pathways has shown that the following pathways are over-represented: innate immune system, interleukin-36 pathway, keratinization, gap junction trafficking and regulation, post-translational modification: synthesis of GPI (Glycosylphosphatidylinositol) -anchored proteins, sphingolipid metabolism and neutrophil degranulation (Additional file 6). The gene ontology (GO) analysis revealed that 114 biological processes were upregulated in the MeDOC group, while 192 were downregulated. The upregulated GO Biological Processes (GO BP) include i.e. epithelial cell differentiation; epidermis development; organonitrogen compound, small molecule metabolic, lipid metabolic process and catabolic process; homeostatic process; regulation of water loss via skin; peptide cross-linking; proteolysis; secretion; cell death; oxidation-reduction process, generation of precursor metabolites and energy, transport. Conversely, the following GO BP were downregulated: locomotion; cell adhesion and motility; developmental process; cell differentiation including positive regulation of cell differentiation; extracellular structure organization; anatomical structure morphogenesis; cell-cell signalling and communication; response to endogenous stimulus; cilium organization; regulation of transport including positive regulation of transport and cation transport. See Additional file 7 for further data.

### Total overview, B- top 50 DEGs

**Fig. 2:**
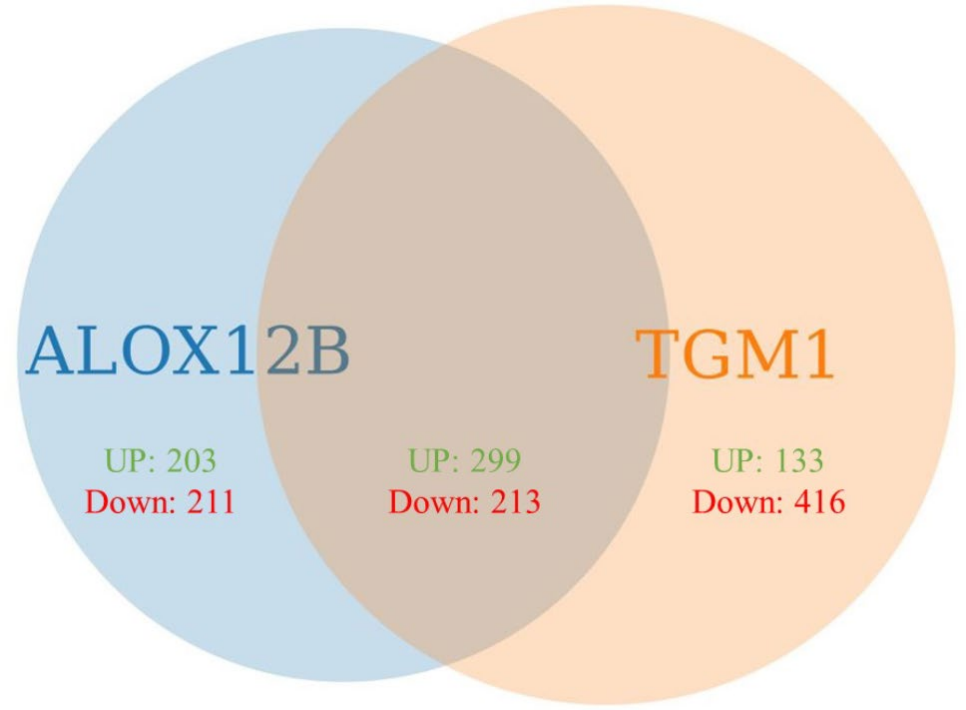
Venn Diagram showing DEGs identified in *ALOX12B* patients vs. controls and *TGM1* patients versus control. UP –upregulated DEGs, Down – downregulated DEGs

Considering the fact that patients with *TGM1* and *ALOX12B* variants were mostly represented in our MeDOC group, we performed the *TGM1* patients vs. controls and *ALOX12B* patients vs. controls analysis and identified 1061 and 926 DEGs, respectively. Next, we compared these two sets of DEGs and found that 512 DEGs were common for *TGM1* and *ALOX12B* groups (Fig. 2). However, when *ALOX12B* vs. *TGM1* DEGs were compared, only 8 were found to be significant, showing that the similarity between these two sets was indeed high.

In terms of Reactome pathways overrepresentation testing, the general pattern was similar to that observed in combined MeDOC samples vs. controls with slight differences only, e.g. “sphingolipid metabolism and gap junction trafficking and regulation” were reported for *ALOX12B* group, while “chemokine receptors bind chemokines” in *TGM1* patients (data not shown).

### Lipid analyses

We performed fatty acid (FAs) profile in epidermal scrapings of patients with ichthyosis caused by pathogenic variants in *ALOX12B* and *TGM1*. Since the sampling site seems to have an effect on the FAs profile (the FAs profile of the back differed from that of the arm), to eliminate the sample site bias we performed two distinct analyses for scrapings taken from arm and back (Tables 5 and 6).

**Table 5:** Composition of selected fatty acids in skin of ichthyosis patients with *ALOX12B* vs. *TGM1* pathogenic variants (arm)

AGE	29.7 ± 7.23	40.0 ± 14.1	0.479
FATTY ACIDS	*ALOX12B* patients	*TGM1* patients	p
16:0	21.6 ± 6.35	16.8 ± 4.65	0.434
18:0	7.33 ± 1.62	12.1 ± 2.79	0.086
20:0	0.98 ± 0.09	1.52 ± 0.17	**0.016**
22:0	1.42 ± 0.29	2.62 ± 0.35	**0.025**
24:0	4.42 ± 0.99	9.40 ± 2.46	**0.045**
26:0	3.52 ± 1.10	7.53 ± 2.62	0.088
28:0	2.42 ± 1.06	4.49 ± 2.91	0.317
30:0	0.60 ± 0.37	0.88 ± 0.68	0.584
32:0	0.04 ± 0.03	0.09 ± 0.07	0.370
other ECFA	5.03 ± 1.21	4.07 ± 2.10	0.548
**ECFA**	**47.4 ± 5.65**	**59.55 ± 0.27**	0.063
21:0	0.14 ± 0.07	0.19 ± 0.05	0.476
23:0	0.56 ± 0.25	1.04 ± 0.52	0.243
25:0	1.15 ± 0.25	2.21 ± 0.79	0.104
27:0	0.60 ± 0.27	1.26 ± 0.85	0.270
29:0	0.31 ± 0.15	0.52 ± 0.41	0.443
31:0	0.03 ± 0.02	0.05 ± 0.04	0.574
other OCFA	3.35 ± 0.35	3.08 ± 0.43	0.485
**OCFA**	**6.14 ± 0.87**	**8.34 ± 2.23**	0.200
other SFA	3.75 ± 0.77	2.84 ± 0.40	0.233
**TOTAL SFA**	**57.3 ± 5.30**	**70.7 ± 1.56**	**0.045**
16:1	8.07 ± 1.19	3.94 ± 2.80	0.096
18:1	25.6 ± 4.32	17.9 ± 2.64	0.116
19:1	0.02 ± 0.01	0.04 ± 0.01	0.282
20:1	0.25 ± 0.05	0.47 ± 0.16	**0.014**
22:1	0.04 ± 0.02	0.12 ± 0.01	**0.027**
24:1	0.05 ± 0.03	0.19 ± 0.03	0.220
26:1	0.03 ± 0.01	0.08 ± 0.03	0.282
other MUFA	1.67 ± 0.31	0.70 ± 0.61	0.228
**TOTAL MUFA**	**35.7 ± 4.24**	**23.4 ± 0.66**	**0.031**
16:2n6	0.01 ± 0.004	0.01 ± 0.00	0.219
LA	6.87 ± 1.28	5.51 ± 1.00	0.300
ARA	0.03 ± 0.000	0.08 ± 0.06	0.192
DGLA	0.02 ± 0.006	0.06 ± 0.01	**0.015**
EDA	0.03 ± 0.02	0.09 ± 0.05	0.165
AdA	0.01 ± 0.000	0.02 ± 0.000	0,200*
**PUFA n6**	**6.96 ± 1.29**	**5.77 ± 0.88**	0.345
ALA	0.02 ± 0.006	0.03 ± 0.01	0.495
EPA	0.03 ± 0.02	0.05 ± 0.04	0.463
DPA	0.02 ± 0.01	0.02 ± 0.01	0.789
DHA	0.02 ± 0.01	0.02 ± 0.01	0.870
**PUFA n3**	**0.08 ± 0.01**	**0.11 ± 0.03**	0.219

Value is mean ± SD. Content of FA given as a percentage (%). p–value Student’s t–test, * p–value Mann–Whitney Rank Sum Test. AdA – adrenic acid (22:4 n6); ALA – α-linolenic acid (18:3 n3); ARA – arachidonic acid (20:4 n6); DGLA –dihomo-γ-linolenic acid (20:3 n6); DHA – docosahexaenoic acid (22:6 n3); DPA – docosapentaenoic acid (22:5 n3); EDA – eicosadienoic acid (20:2 n6); EPA – eicosapentaenoic acid (20:5 n3); HDA – hexadecadienoic acid (16:2 n6), LA – linoleic acid (18:2 n6); ECFA – even chain fatty acids; MUFA – monounsaturated fatty acids. OCFA – odd chain fatty acids; PUFA – polyunsaturated fatty acids; SFA – saturated fatty acids. Boldface - major groups of fatty acid

**Table 6:** Composition of selected fatty acids in skin of ichthyosis patients with *ALOX12B* vs. *TGM1 *pathogenic variants (back)

age	17.0 ± 2.83	34.0 ± 21.1	0.378
**FATTY ACIDS**	*ALOX12B patients*	*TGM1 patients*	**p**
16:0	26.9 ± 4.77	14.3 ± 2.28	0.078
18:0	8.06 ± 0.64	13.8 ± 5.61	0.290
20:0	0.80 ± 0.03	1.86 ± 0.86	0.225
22:0	0.85 ± 0.05	3.49 ± 1.10	0.077
24:0	2.96 ± 0.78	11.7 ± 3.01	0.058
26:0	2.25 ± 0.78	7.69 ± 2.99	0.131
28:0	1.53 ± 0.28	4.78 ± 1.45	0.090
30:0	0.33 ± 0.13	0.95 ± 0.08	**0.028**
32:0	0.05 ± 0.02	0.08 ± 0.04	0.412
other ECFA	7.39 ± 0.42	3.11 ± 0.52	**0.012**
**ECFA**	**51.1 ± 2.98**	**61.7 ± 0.90**	**0.040**
21:0	0.06 ± 0.000	0.37 ± 0.17	0.123
23:0	0.33 ± 0.11	1.74 ± 0.42	**0.045**
25:0	0.80 ± 0.42	2.74 ± 0.81	0.096
27:0	0.37 ± 0.15	1.23 ± 0.40	0.105
29:0	0.21 ± 0.13	0.51 ± 0.000	0.085
31:0	0.06 ± 0.06	0.08 ± 0.05	0.759
other OCFA	6.25 ± 1.71	2.90 ± 1.63	0.333*
**OCFA**	**8.06 ± 2.59**	**9.55 ± 3.39**	0.671
other SFA	5.21 ± 0.02	1.55 ± 0.04	**0.000**
**TOTAL SFA**	**64.4 ± 0.37**	**72.8 ± 4.26**	**0.108**
16:1	16.1 ± 0.42	3.07 ± 0.30	**0.001**
18:1	12.7 ± 1.68	18.6 ± 6.12	0.321
19:1	0.01 ± 0.00	0.04 ± 0.01	0.095
20:1	0.14 ± 0.04	0.39 ± 0.21	0.231
22:1	0.02 ± 0.00	0.10 ± 0.08	0.314
24:1	0.03 ± 0.01	0.24 ± 0.23	0.331
26:1	0.03 ± 0.02	0.06 ± 0.04	0.406
other MUFA	3.25 ± 0.98	0.59 ± 0.10	0.062
**TOTAL MUFA**	**32.3 ± 0.22**	**23.1 ± 5.95**	**0.160**
16:2n6	0.01 ± 0.000	0.01 ± 0.005	0.423
LA	3.25 ± 0.56	3.85 ± 1.69	0.681
ARA	0.01 ± 0.000	0.07 ± 0.000	0.333*
DGLA	0.02 ± 0.01	0.02 ± 0.01	1.000*
EDA	0.01 ± 0.005	0.05 ± 0.04	0.267
AdA	0.01 ± 0.005	0.03 ± 0.02	0.353
**PUFA n6**	**3.29 ± 0.56**	**4.01 ± 1.72**	**0.633**
ALA	0.02 ± 0.01	0.03 ± 0.01	0.667*
EPA	0.01 ± 0.000	0.02 ± 0.000	0.333*
DPA	0.02 ± 0.01	0.04 ± 0.01	0.155
DHA	0.02 ± 0.01	0.04 ± 0.01	0.333*
**PUFA n3**	**0.07 ± 0.01**	**0.13 ± 0.01**	**0.028**

Value is mean ± SD. Content of FA given as a percentage (%). p–value Student’s t–test, * p–value Mann–Whitney Rank Sum Test. AdA – adrenic acid (22:4 n-6); ALA – α-linolenic acid (18:3 n-3); ARA – arachidonic acid (20:4 n-6); DGLA –dihomo-γ-linolenic acid (20:3 n-6); DHA – docosahexaenoic acid (22:6 n-3); DPA – docosapentaenoic acid (22:5 n-3); EDA – eicosadienoic acid (20:2 n-6); EPA – eicosapentaenoic acid (20:5 n-3); HDA – hexadecadienoic acid (16:2 n-6), LA – linoleic acid (18:2 n-6); ECFA – even chain fatty acids; MUFA – monounsaturated fatty acids. OCFA – odd chain fatty acids; PUFA – polyunsaturated fatty acids; SFA – saturated fatty acids. Boldface - major groups of fatty acid

The largest differences were observed for the very long chain fatty acids (fatty acids with carbon chain length ≥ C20 - FAs ≥ C20, VLCFAs; ), the very long-chain monounsaturated fatty acids (VLC-MUFAs, FAs ≥ C20:1) and the n6 polyunsaturated fatty acids (n6 PUFAs) (Table 5). Due to the small sample size, many FAs only showed an upward or downward trend (Tables 5 and 6). Differences were found in ultra-long chain fatty acids (ULCFA, FAs ≥ C28) and n3 polyunsaturated fatty acids (n3 PUFAs). Interestingly, the palmitoleic acid content was 5 times lower in the *TGM1* sample compared to *ALOX12B* (Table 6).

## Discussion

### General summary

In this study, we have conducted comprehensive molecular analysis of a cohort of 265 Polish patients with cornification disorders. Novel pathogenic variants and modifying variants were detected in MeDOC patients. For part of the patients, we also preformed transcriptome analysis and described characteristics of *ALOX12B* vs. *TGM1* deficient epidermis on mRNA and lipid levels.

In the cases analysed by NGS (245) the diagnostic yield of 87% was achieved, a value similar to the diagnostic efficacy obtained by others [12]. A higher detection rate of 89% was obtained in a small subgroup of X-linked ichthyosis patients, selected by an X-linked inheritance pattern, in whom only the *STS* MLPA test was performed.

The majority of patients were sent from different clinical centres across Poland and their clinical evaluation was limited or not available, making the unbiased, broad NGS-gene panel the method of choice. However, as the clinical data were not fully available, we had to trust the suspicion of MeDOC made by the referring clinician and therefore could not assess with certainty if in the 13% of unsolved cases, the right diagnostic methods were used. For example Nagtzaam et al., reported that in Dutch population in 20% of cases with clinical suspiction of X-linked ichthyosis, the genetic analysis showed variants in the other gene(s) than *STS*, leading to diagnosis change [13].

Based on the presented results, we can conclude that in the referred population ARCI patients were mostly represented, (126/254, 49%). In 56/126 (45%) cases, the *ALOX12B* gene was involved, making it the most common cause of ARCI in the tested population, while in 33/126 (26%) and 18/126 (14%) of cases the *TGM1* and *ALOXE3* genes were causal, respectively.

The overrepresentation of *ALOX12B* patients was also noticed among Czech ARCI patients, where 18/47 (38%) probands had *ALOX12*B pathogenic variants [14] and among Middle-Eastern ARCI patients of Muslim origin – 26% [15]. This is in contrast to several other European studies, that indicated the *TGM1* variants as the ARCI leading cause, e.g. in Austria, Scandinavia, Galicia [12, 15–17]. These discrepancies could at least partially be explained by the high abundancy of recurrent *ALOX12B* and *ALOXE3* variants in the Polish and regional (Czech) population [14]. Nevertheless, in the future, the availability of more population-specific genomic data will enable more precise estimations regarding variant frequency.

A large multicenter study on 224 *ALOX12B* and *ALOXE3* patients of various ethnicities has shown that the p.Tyr521Cys variant accounts for 22% of all *ALOX12B* alleles [18]. In our group however, this variant was detected in 45% (52 of 115) of *ALOX12B* alleles (including the one allele found as a secondary finding). In Austrian and Czech cohorts this variant was also frequent and present in 6/14 (42%) and 10/36 (28%) of *ALOX12B* alleles, respectively [12, 14]. Although the origin of the p.Tyr521Cys variant is not known, our results suggest that a founder effect may have contributed to its high prevalence in Poland.

Of note, another frequent variant in *ALOX12B*: p.Ala597Glu was found in 11/115 alleles (9.5%) in our cohort, similarly to Czech patients (4/36 alleles – 10%), while in multicentre studies mentioned above, it was found in 12/282 (4%) alleles, confirming its regional higher prevalence [14, 18]. For *ALOXE3*, the most prevalent variant: p.Arg234Ter was detected in 22/37 (59%) alleles and 9/18 (50%) in Polish and Czech cohorts, respectively. Among various ethnicities this variant was present in 21% of *ALOXE3* alleles. Interestingly, the p.Pro630Leu was present in our population only once, while in a multicentred and Czech cohort it was frequent (40% and 28% of *ALOXE3* alleles, respectively) [14, 18].

The *TGM1* results revealed, beside two well-known recurrent pathogenic variants: p.Arg126His and p.Val379Leu, an additional one: duplication of exons 10–14 (c.(1402 + 1_1401-1)_(2225 + 1_2226-1)dup), found in 9 probands. According to the HGMD database (v. Professional 2023.3), this variant was described only once in the literature before, but was detected using in silico NGS analysis in a single patient and its presence was not confirmed by DNA analysis [17]. We performed DNA quantitative tests in probands and their parents, which confirmed that the duplication was present (manuscript under preparation).

### Additional variants

In 23 cases of 245 analysed by NGS (9%), we revealed additional variants in genes encoding proteins involved in epidermal barrier formation. Most of them were known pathogenic/likely pathogenic ones, however few were novel and few were VUS. There is no consensus in Poland and Europe as to the genetic testing and reporting of single allele incidental findings in autosomal recessive disorders. For example, in Poland carriership of pathogenic/likely pathogenic variants in the genes included in the phenotype-related panels are reported for the purpose of genetic counselling, while in the Netherlands often only causative variants are, and carrier status is not a part of the diagnostic result. Furthermore, there is an open question whether the single allele variants influence the phenotype. This aspect is intriguing in particular, considering that in 10/23 cases, the additional gene was *FLG*. This gene encodes filaggrin, the multitasking protein involved in epidermal differentiation, barrier formation and moisturising. *FLG* defects lead to ichthyosis vulgaris (IV), an autosomal semidominant condition with incomplete penetrance (83–96%) and variable expressivity [19, 20]. Biallelic pathogenic variants in *FLG* are associated with a relatively severe phenotype, however the vast majority of patients have a mild phenotype of IV and/or symptoms of atopy due to heterozygous mutation in FLG [20–24].

It has already been anecdotally observed that *FLG* variants exaggerate the symptoms of X-linked ichthyosis [25–29]. Moreover, in a Dutch cohort of 109 male patients with clinical suspicion of X-linked ichthyosis (XLI), *FLG* variants were concomitant with an *STS* pathogenic variant in 4% of cases [13]. Another report showed a *FLG* variant in a patient with ARCI caused by *PNPLA1* pathogenic variants, however it is impossible to say if and how the phenotype was influenced by filaggrin defect [30]. It seems that *FLG* variants are not well recognized or described as incidental findings in ichthyosis types other than X-linked ichthyosis. It is safe to assume that the frequency of *FLG* pathogenic variants in the general European population is around 4–7% [20, 31]. However, in the available reports on ichthyosis cohorts, the presence of *FLG* pathogenic variants is either not reported or was not investigated.

In the remaining cases, we detected pathogenic variants in the other genes, that can be causative for different types of autosomal recessive ichthyosis (ARCI). Importantly, six of them were nonsense variants, meaning that only 50% of protein would be produced, provided the second allele is normal. As many of the ARCI subtypes share the same pathways/processes in the epidermis such an haploinsufficiency of the other protein might hypothetically have some influence on the phenotype. Such observations have already been made in other genodermatoses, e.g. epidermolysis bullosa [32–34]. Therefore further studies are needed to verify if this has clinical implications in ichthyosis.

### Transcriptomics and lipidomics

The transcriptome analysis of 18 patients revealed overexpression of genes encoding proteins involved in immunity, epidermis development, differentiation and signalling. In contrary, downregulation of those involved in cell adhesion and motility, developmental process and differentiation, extracellular structure organization, signalling and communication was shown.

Recent transcriptomic analyses showed the Th17/Th22 mediated immune skewing in different clinical subtypes of ichthyosis [35, 36]. In accordance with their studies, the Th17/Th22 markers were also upregulated in our group. Six of them were among the top 50 Differentially Expressed Genes (DEG) (*S100A9*,* S100A8*,* S100A7*,* SERPINB3*,* SERPINB4*,* S100A7A*), but the overall number of upregulated genes involved in Th17/Th22 immune response included also *IL36G*,* IL36RN*,* IL26*,* KLK10*,* EPN3*,* PI3*,* VNN3* and others. Importantly, despite general convergent gene expression profile, some discrepancies regarding individual genes are noticeable. For example, Kim detected IL17A/C, IL22 and IL-23R expression using RNA-seq, that was below the threshold in Malik analysis based on microarrays [36]. In our group, only *IL17C* and *IL22RA1* (Subunit Alpha1 for IL22) were upregulated, although in *IL22RA1* logFC was 1.6. This may reflect individual differences, methodological issues and heterogeneity of ichthyoses, as discussed below. Nevertheless, genes involved in cornification and barrier formation were concordantly upregulated in this and previous studies.

Furthermore, in contrast to Kim et al., who reported downregulation of lipid metabolism genes, we found upregulation of several genes involved in unsaturated fatty acid biosynthetic, oxoacid and lipid metabolic processes. However, when we compared our results with those of Kim, it turned out that of 277 genes upregulated in our group, around 40 were also upregulated in their patients and only a few were assigned as downregulated [36]. Moreover, a set of genes related to lipid biosynthesis was shown to be upregulated in *TGM1*-ARCI patients, as reported by Zhang et al. [37]

In order to have further insight into this issue, we compared the expression profile narrowing to two patients subgroups: *ALOX12B*-deficient (*n* = 5) and *TGM1*-deficient (*n* = 7) patients. Those subgroups were the most numerous in our group of patients. The proteins encoded by both genes are involved in production and formation of the corneocyte lipid envelope. Specifically, *ALOX12B* encodes arachidonate 12-lipoxygenase which is a key enzyme processing arachidonic acid (20:4n–6) during synthesis of barrier lipids, while transglutaminase 1 (*TGM1*) mediates the cross-linking of proteins in the corneocyte protein envelope and the attachment of the corneocyte lipid envelope [38].

Although differences between the number of upregulated and downregulated genes were shown when *ALOX12B*-deficient vs. controls and *TGM1*-deficient vs. controls DEGs sets were compared, we haven’t found DEGs between *ALOX12B*-deficient and *TGM1*-deficient patients, emphasizing that the expression pattern in those two groups are highly similar. In contrast, when we compared the fatty acids (FAs) profile in the epidermis taken from lesional skin of ALOX12B-deficient and TGM1-deficient patients, the differences in content of selected groups of FAs were visible. Recently we analysed the FAs profile in normal epidermis, which showed the differences in FA distribution with respect to age and skin location [10]. Therefore, the results of MeDOC patient analyses were narrowed with respect to collection site. We showed that the content of C16:1 in *TGM1* was lower than in *ALOX12B* patients (inactive ALOX12B impairs processing of lipids biosynthesis). Conversely, the number of long chain FAs was higher in *TGM1* patients. Though the tendency is visible, the statistical significance was achieved in only certain classes of FAs, probably due to small group sizes.

Previous studies on atopic dermatitis and the Netherton syndrome have already shown that the level of C16-18 adversely corresponds with epidermal barrier function [39, 40]. Importantly, it has been proposed, that the altered ratio of mono – vs. saturated long chain fatty acids affects production of substrates necessary for ceramide synthesis [41]. Moreover, in vitro studies provided data showing that increase in FAs and/or monounsaturated fatty acids (MUFA) content influences lipid organisation and, consequently, barrier permeability [40]. Our results also focus on this aspect showing that in *ALOX12B* patients, the amount of total even chain fatty acids (ECFA), odd chain fatty acids (OCFA) and saturated fatty acids (SFA) were diminished compared to the *TGM1* group, with the exception of MUFA that was increased. Although detailed data on lipid composition in MeDOC skin are limited, it is well known that the ratio of main lipid types, as well as their content and organisation, vary in different ichthyosis types [42]. Whether phenotypic differences between patients with defects in *ALOX12B* and *TGM1* genes are directly related to the different lipid levels and composition is highly intriguing. Unfortunately, due to limited data, we are currently unable to comment on this aspect. Of note, however, we are the first to publish comparative data on FA in epidermis of patients with ARCI. Hence, providing further insights into the complexity of lipid homeostasis in the ARCI epidermis, we also provoke novel (yet unanswered) questions.

### Limitation of the study

We analysed large cohort of patients with different types of MeDOC, the clinical data in most cases were highly limited, though. In majority of cases the referral doctors indicated only “ichthyosis” or “keratoderma” on the referral form. Importantly, the material was referred to our laboratories by over 70 clinicians (geneticists, dermatologists, neonatologists) from over 30 different clinical canters across country. Therefore, in frame of this manuscript it was not possible to provide clinical descriptions. Importantly, MeDOCs are group of rare diseases, therefore the most valuable phenotypic evaluations, enabling the assessment of clinical nuances, would be those performed in specialized reference canters. Nevertheless, few cases of MeDOC patients presented in this article were phenotypically characterised before (as indicated in Additional file 3) [8, 11, 43].

Although the genetic basis of MeDOC are generally well established, the functional consequences of genetic defects in barrier formation genes is less recognised. Currently more tools are available for robust wide-ranging biochemical research, however there are still several limitations of such studies. Accordingly, there were also limitations of our study. Firstly, we encountered difficulties in collecting epidermal samples from healthy volunteers. We struggled to match age, sex and biopsy site in our control group. Therefore, the majority of control skin was mostly obtained during bariatric surgery from normal skin of feminine abdomen without clinical symptoms of MeDOC. Also genotyping was not performed in the control group.

## Conclusions

In conclusion, our results are the first compilation of genetic analysis of a large cohort of 265 patients with inherited epidermal barrier dysfunction of Polish origin. We report novel variants, and show that copy number variations are among the major group of molecular defects not only in *STS*, but also in *TGM1* and *SPINK5* genes. This significantly broadens the knowledge about molecular pathology in these disorders. Furthermore, we provide evidence that the pathogenic variants in more than one gene are detected in several patients, which should be further investigated in the context of phenotypic modifiers. In terms of functional studies, our data add an unique characteristic of *ALOX12B* vs. *TGM1* deficient epidermis on mRNA and lipid levels.

## Electronic supplementary material

Below is the link to the electronic supplementary material.

Supplementary Material 1

Supplementary Material 2

Supplementary Material 3

Supplementary Material 4

Supplementary Material 5

Supplementary Material 6

Supplementary Material 7

## Data Availability

The datasets supporting the conclusions of this article are available in the repository of Institute of Mother and Child in Warsaw and are available from the corresponding author upon reasonable request.

## Abbreviations

ACMG: American College of Medical Genetics
ARCI ALA: α-linolenic acid (18:3 n3)
DEG: Differentially Expressed Genes
ECFA: Even chain fatty acids
FA: Fatty acids
FDR: False Discovery Rate
GO BP: GO Biological Processes
GO: Gene ontology
LogFC: log fold change
MeDOC: Ichthyoses or Mendelian Disorders of Cornification
MUFA: monounsaturated fatty acids
n3 PUFAs: n3 polyunsaturated fatty acids
n6 PUFAs: n6 polyunsaturated fatty acids
OCFA: Odd chain fatty acids
PCA: Principal component analysis
PPK: Palmolpantar keratoderma ()
PUFA: Polyunsaturated fatty acids
SFA: Saturated fatty acids
ULCFA: Ultra-long chain fatty acids (FAs ≥ C28)
VLCFAs: Very long chain fatty acids (FAs ≥ C20)
VLC-MUFAs: Very long-chain monounsaturated fatty acids (FAs ≥ C20:1)
