# Supplementary material for: Molecular analysis of inherited disorders of cornification in polish patients show novel variants and functional data and provokes questions on the significance of secondary findings
Source: Orphanet J Rare Dis. 2024 Nov 5;19:413. doi: 10.1186/s13023-024-03395-4 (PMC11536877; doi:10.1186/s13023-024-03395-4)
Supplement: Supplementary file 1 — Supplementary Material 1 [file 13023_2024_3395_MOESM1_ESM.docx]

Additional file 1.

The characteristics of samples used in the functional analyses

1. The general characteristics of patients and controls samples

| **Number** | **SAMPLE ID** | **AGE** | **SEX** | **skin sample site** | **genotype** |
| --- | --- | --- | --- | --- | --- |
| P1 | **11** | 4 | F | tight | *ALOXE3*:p.Arg234Ter/Pro630Leu |
| P2 | **19** | 25 | F | arm | *ALOX12B*: p.Ty521Cys/p.Tyr521Cys |
| P3 | **43** | 18 | M | arm | *TGM1*: p.Val379Leu/Trp193Ter |
| P4 | **56** | 17 | F | arm | *ALOXE3*:p.Arg234Ter/p.Arg234Ter;  *DSP*: p.Arg1400Ter/- |
| P5 | 41 | 30 | F | arm | *TGM1*:p.Val379Leu/p.Glu497Val |
| P6 | **54** | 26 | F | arm | *ALOX12B*: p.Tyr521Cys/p.Phe485Ser |
| P7 | **24** | 10 | M | arm | *STS*: c.(?_-1)_(*1_?)del |
| P8 | **58** | 13 | F | arm | *TGM1*: p.Trp193Ter/p.Trp193Ter |
| P9 | **57** | 7 | M | arm | *SPINK5*: p.Phe606Leu/c.1816_1820+21delinsCT |
| P10 | **39** | 19 | F | back | *ALOX12B*: p.Tyr521Cys/Pro422Leu  *FLG*: p.Ser761CysfsTer36/-, |
| P11 | **53** | 37 | M | knee | *LOR*:c.639_642dup (p.Thr215GlyfsTer122)/- |
| P12 | **45** | 49 | M | arm, back | *TGM1*: Arg126His/Arg126His |
| P13 | **55** | 9 | M | arm | *TGM1*:p.Arg315Cys/ex10-14dup |
| P14 | **52** | 15 | F | back | *ALOX12B*:p.Tyr521Cys/p.Tyr521Cys |
| P15 | **36** | 9 | M | back | *TGM1*:p.Arg126His/c.877-2A>G |
| P16 | **10** | 50 | M | arm | *TGM1*: p.Arg126His/p.Arg126His |
| P17 | **34** | 30 | F | unknown | *ABCA12*: p.Asn1380Ser/p.Asn2034Asp  *FLG*:p.Arg2447Ter/- |
| P18 | **38** | 19 | F | unknown | *ALOX12B*:p.His158CysfsTer20 (c.467_470dup)/p.Tyr521Cys |
| C1 | **23** | 3 | male | abdomen |  |
| C2 | **42** | 9 | F | tight |  |
| C3 | **21** | 37 | F | arm |  |
| C4 | **26** | 10 | U | back |  |
| C5 | **20** | 50 | F | breast |  |
| C6 | **40** | 52 | F | breast |  |
| C7 | **30** | 52 | F | breast |  |
| C8 | **51** | nd | nd | tight |  |
| C9 | **27** | 39 | M | knee |  |
| C10 | **32** | nd | nd | nd |  |
| C11 | **35** | 50 | F | abdomen |  |
| C12 | **50** | 56 | F | abdomen |  |
| C13 | **37** | 56 | F | abdomen |  |
| C14 | **33** | 30 | F | abdomen |  |
| C15 | **44** | 65 | F | abdomen |  |
| C16 | **22** | 65 | F | abdomen |  |
| C17 | **29** | 42 | F | abdomen |  |
| C18 | **49** | 3 | nd | tight |  |
| C19 | **48** | 3 | M | tight |  |
| C20 | **25** | 4 | M | tight |  |
| C21 | **31** | 51 | F | breast |  |
| C22 | **28** | nd | F | arm |  |

1. The clinical characteristics of selected patients with *ALOX12B* and *TGM1* pathogenic variants

| **gene** | *ALOX12B* | | | | | *TGM1* | | | | |
| --- | --- | --- | --- | --- | --- | --- | --- | --- | --- | --- |
| **age range** | 18-30 | 18-30 | 18-30 | 18-30 | 18-30 | 30-60 | 18-30 | <18 | <18 | 30-60 |
| **sex** | f | f | f | f | f | m | m | m | m | m |
| **itch (NRS scale [0-10])** | 7 | 0 | 6 | 3 | 6 | 7 | 6 | 7 | 6 | 7 |
| **pain (NRS scale [0-10])** | 0 | 0 | 0 | 0 | 0 | 0 | 4 | 5 (occasionally) | 0 | 4 (occasionally) |
| **localisation of itch/pain** | whole body | - | whole body | back | forehead, thighs, forearms and chest | itching, intensifies after scratching and spreads to the entire body | hands and feet mainly | mainly on the head, abdomen and shoulder blades, rarely on the legs | whole body , exept for palmoplantar regions | whole body |
| **scaling** | ***y*** | n | ***y*** | ***y*** | ***y*** | ***y*** | ***y*** | ***y*** | ***y*** | ***y*** |
| **localisation of scaling** | whole body | pretibia | whole body | whole body (less on torso and arms, more on legs and face) | whole body | whole body (less on the head, thighs and buttocks) | whole body | whole body | head | whole body |
| **scales color** | transparent, white, turns brown mainly in summer in certain areas | n | slightly brown | white | white | turns into brown | turns into brown | transparent, white, turns brown in certain areas | bright, turns into brown | nd |
| **palmar and/or plantar keratoderma** | ***y*** | ***y*** | ***y*** | ***y*** | ***y*** | n | ***y*** | ***y*** | n | ***y*** |
| **erythema** | ***y*** | ***y*** | n | ***y*** (mainly face and body parts exposed) | n | n | n | ***y*** (only after scratching) | n | n |
| **palmar hyperlinearity** | ***y*** | ***y*** | ***y*** | n | ***y*** | ***y*** | n | ***y*** | n | ***y*** |
| **maceration during short contact with water** | n | n | ***y*** | n | n | ***y*** (slight) | nd | ***y*** (slight) | n | n |
| **knuckle pads** | n | n | nd | n | n | ***y*** | ***y*** | n | n | n |
| **keratosis pilaris** | ***y*** | ***y*** | n | n | n | ***y*** | ***y*** | ***y*** |  | ***y*** |
| **Athopy** | | | | | | | | | | |
| **eczema** | n | n | n | n | ***y*** | n | n | n | n | n |
| **asthma** | n | n | n | n | n | n | n | n | n | n |
| **allergic rhinitis** | n | ***y*** | n | n | n | n | ***y*** | n | ***y*** | ***y*** |
| **Other** | | | | | | | | | | |
| **nail changes** | n | n | n | n | n | ***y*** | ***y*** | ***y*** | n | n |
| **tooth changes** | n | n | n | n | n | n | n | n | n | ***y*** |
| **hair changes** | n |  | n | n | dense, but thinned out due to shedding | sparse hair | ***y*** | sparse hair with regional alopecia | n | sparse hair |
| **eyebrows, eyelashes, body hair** | n | n | normal | slightly reduced | normal | reduced | reduced | normal (prepuberty) | normal (prepuberty) | reduced |
| **Hypohidrosis** | ***y*** | n | n | ***y*** | ***y*** | ***y*** | ***y*** | ***y*** | ***y*** | ***y*** |
| **excessive sweating** | n | n | n | n | n | ***y*** (limited to less scaling regions) | n | n | n | n |
| **Presence at birth** | | | | | | | | | | |
| **colloidon baby** | ***y*** | ***y*** | ***y*** | ***y*** | ***y*** | nd | ***y*** | ***y*** | n | nd, but born severely affected |
| **Harlekin** | n | n |  | n | ***y*** | nd | n | n | n | nd |
| **erythroderma** | ***y*** | ***y*** | ***y*** | ***y*** | ***y*** | n | nd (but present in affected his sister) | t | n | nd |
| **blisters** | n | n | n | n | ***y*** | n | n | n | n | nd |
| **hyperkeratosis** | ***y*** | ***y*** | ***y*** | n | ***y*** | ***y*** | ***y*** | ***y*** | ***y*** | nd |
| **ectropion** | ***y*** | ***y*** | n | n | n | n | ***y*** | ***y*** | ***y*** | nd |
| **OTHER** | | | | | | | | | | |
| **descent of the testicles** | - | - | - | - | - | n | ***y*** | n | n | n |
| **epilepsy** | n | n | n | n | n | n | n | n | n | n |
| **growth problems** | n | n | n | n | n | n | n | ***y*** | n | n |
| **delay in motor development** | n | n | n | n | n | n | n | n | n | n |
| **speech/language delay** | n | n | n | n | n | n | n | n | n | nd |
| **sight problems** | n | n | n | n | n | ***y*** | ***y*** | ***y*** | ***y*** | n |
| **hearing problems** | n | n | n | n | ***y*** | ***y*** | ***y*** | ***y*** | n | ***y*** |
| **frequent infections** | ***y*** (in childhood) | n | n | n | ***y*** | n | n | n | n | n |
| **vitamin D deficiency** | n | *nd* | n | ***y*** | n | nd | n | n | n | t |
| **heart defects** | n | n | n | n | n | n | n | ***y*** | n | n |
| **defects of the skeletal system** | n | ***y*** | n | n | n | n | n | n | n | n |
