# Supplementary material for: Molecular analysis of inherited disorders of cornification in polish patients show novel variants and functional data and provokes questions on the significance of secondary findings
Source: Orphanet J Rare Dis. 2024 Nov 5;19:413. doi: 10.1186/s13023-024-03395-4 (PMC11536877; doi:10.1186/s13023-024-03395-4)
Supplement: Supplementary file 2 — Supplementary Material 2 [file 13023_2024_3395_MOESM2_ESM.docx]

Additional file 2

Summary of variant distribution among 265 patients

| **Gene** | **number of variants (n=150)** | **total number of alleles** (n=415)** |
| --- | --- | --- |
| *ALOX12B** | 22 | 115 |
| *TGM1** | 23 | 68 |
| *FLG* | 8 | 39 |
| *ALOXE3** | 15 | 37 |
| *STS* | 2 | 27 |
| *SPINK5* | 12 | 20 |
| *ABCA12** | 14 | 19 |
| *KRT10* | 7 | 15 |
| *KRT9* | 3 | 14 |
| *NIPAL4** | 3 | 11 |
| *KRT1* | 7 | 6 |
| *CYP4F22** | 3 | 6 |
| *SLC27A4** | 3 | 4 |
| *PNPLA1** | 2 | 4 |
| *GJB2* | 3 | 3 |
| *KRT2* | 3 | 3 |
| *AAGAB* | 1 | 1 |
| *DSP* | 2 | 2 |
| *ERCC2* | 2 | 2 |
| *ALDH3A2* | 1 | 2 |
| *CERS3** | 1 | 2 |
| *LORICRIN* | 1 | 2 |
| *POMP* | 1 | 2 |
| *DSG1* | 1 | 1 |
| *GJA1* | 1 | 1 |
| *GJB3* | 1 | 1 |
| *GJB4* | 1 | 1 |
| *KRT16* | 1 | 1 |
| *ST14* | 1 | 1 |
| *ERCC3* | 2 | 2 |
| *GTF2H5* | 1 | 1 |
| *VPS33B* | 1 | 1 |
| *WNT10A* | 1 | 1 |

* Autosomal Recessive Congenital Ichthyosis (ARCI) genes

** Includes all the variants detected during realisation of the study i.e. those identified in the group of patients with causative pathogenic variants found; variants detected as secondary findings and variants detected in patients, in whom only one pathogenic variant in the ARCI-causing gene was detected (data not shown).
