# Supplementary material for: Molecular analysis of inherited disorders of cornification in polish patients show novel variants and functional data and provokes questions on the significance of secondary findings
Source: Orphanet J Rare Dis. 2024 Nov 5;19:413. doi: 10.1186/s13023-024-03395-4 (PMC11536877; doi:10.1186/s13023-024-03395-4)
Supplement: Supplementary file 3 — Supplementary Material 3 [file 13023_2024_3395_MOESM3_ESM.docx]

Additional File 3

Summary of MeDOC genotypes identified in the study with clinical data (n=226)

|  | **GENES and CORRESPONDING PHENOTYPES according to OMIM** | |
| --- | --- | --- |
| No. | GENOTYPE (AND CLINICAL FEATURES OF PATIENTS IF AVAILABLE) | NUMBER OF PATIENTS WITH THE SAME GENOTYPE |
|  | ***AAGAB*** | |
|  | Keratoderma, palmoplantar, punctate type IA (OMIM 148600), AD | |
| 1 | NM_024666.4:c.451+1G>A/- ; p.?/- | 1 |
|  | PPK |  |
|  | ***ABCA12*** | |
|  | Ichthyosis, congenital, autosomal recessive 4A (OMIM 601277), AR  Ichthyosis, congenital, autosomal recessive 4B (harlequin) (OMIM 242500), AR | |
| 1 | NM_173076.2:c.179G>C/c.1803G>A ; p.Arg60Pro/p.Trp601Ter | 1 |
| 2 | NM_173076.2:c.2140C>T/c.2140C>T ; p.Arg714Ter/p.Arg714Ter | 1 |
| 3 | NM_173076.2:c.2638G>C/c.5878C>T ; p.Val880Leu/p.Arg1960Ter | 1 |
| 4 | NM_173076.2:c.4139A>G/c.4951G>A ; p.Asn1380Ser/p.Gly1651Ser | 1 |
| 5 | NM_173076.2:c.4139A>G/c.6100A>G ; p.Asn1380Ser/p.Asn2034Asp | 1 |
| 6 | NM_173076.2:c.4543C>T/c.4543C>T ; p.Arg1515Ter/p.Arg1515Ter | 1 |
| 7 | NM_173076.2:c.6194del/c.5848C>T ; p.Asn2065ThrfsTer3/p.Arg1950Ter | 1 |
| 8 | NM_173076.2:c.758delT/c.6611G>A ; p.Phe253SerfsTer27/p.Arg2204Gln | 1 |
|  | ***ALDH3A2*** | |
|  | Sjogren-Larsson syndrome (OMIM 270200), AR | |
| 1 | NM_001031806.1:c.734A>C/c.734A>C ; p.Asp245Ala/p.Asp245Ala | 1 |
|  | photophobia, delayed motor development, colloidon baby |  |
|  | ***ALOX12B*** | |
|  | Ichthyosis, congenital, autosomal recessive 2 (OMIM 242100), AR | |
| 1 | NM_001139.2:c.1154T>C/c.1562A>G ; p.Val385Ala/p.Tyr521Cys | 1 |
| 2 | NM_001139.2:c.1163C>T/c.1163C>T ; p.Ala388Val/p.Ala388Val | 1 |
| 3 | NM_001139.2:c.1163C>T/c.1579G>A ; p.Ala388Val/p.Val527Met | 1 |
| 4 | NM_001139.2:c.1207C>T/c.1790C>A ; p.His403Tyr/p.Ala597Glu | 1 |
|  | Clinical data available in Pietrzak et al., 2022 |  |
| 5 | NM_001139.2:c.1265C>T/c.1265C>T ; p.Pro422Leu/p.Pro422Leu | 1 |
| 6 | NM_001139.2:c.1265C>T/c.1562A>G ; p.Pro422Leu/p.Tyr521Cys | 3 |
| 7 | NM_001139.2:c.1294C>T/c.1790C>A ; p.Arg432Ter/p.Ala597Glu | 1 |
| 8 | NM_001139.2:c.1454T>C/c.1562A>G ; p.Phe485Ser/p.Tyr521Cys | 2 |
| 9 | NM_001139.2:c.1529A>G/c.1562A>G ; p.Glu510Gly/p.Tyr521Cys | 1 |
| 10 | NM_001139.2:c.1562A>G/c.808A>C ; p.Tyr521Cys/p.Asn270His | 1 |
| 11 | NM_001139.2:c.1562A>G/c.1265C>T ; p.Tyr521Cys/p.Pro422Leu | 1 |
| 12 | NM_001139.2:c.1562A>G/c.1562A>G ; p.Tyr521Cys/p.Tyr521Cys | 12 |
| 13 | NM_001139.2:c.1562A>G/c.1579G>A ; p.Tyr521Cys/p.Val527Met | 1 |
| 14 | NM_001139.2:c.1562A>G/c.1790C>A ; p.Tyr521Cys/p.Ala597Glu | 3 |
| 15 | NM_001139.2:c.1562A>G/c.2094C>A ; p.Tyr521Cys/p.Ser698Arg | 4 |
|  | Clinical data available in Pietrzak et al., 2022 (1 patient) |  |
| 16 | NM_001139.2:c.1562A>G/c.911T>C ; p.Tyr521Cys/p.Leu304Ser | 1 |
| 17 | NM_001139.2:c.1790C>A/c.1790C>A ; p.Ala597Glu/p.Ala597Glu | 1 |
| 18 | NM_001139.2:c.1A>G/c.1562A>G ; p.Met1Val/p.Tyr521Cys | 4 |
| 19 | NM_001139.2:c.1A>G/c.1790C>A ; p.Met1Val/p.Ala597Glu | 1 |
| 20 | NM_001139.2:c.1A>G/c.1A>G ; p.Met1Val/p.Met1Val | 1 |
| 21 | NM_001139.2:c.1A>G/c.2094C>A ; p.Met1Val/p.Ser698Arg | 1 |
| 22 | NM_001139.2:c.2036G>A/c.2060A>G ; p.Arg679His/p.Tyr687Cys | 1 |
|  | Clinical data available in Pietrzak et al., 2022 (1 patient) |  |
| 23 | NM_001139.2:c.2094C>A/c.467_470dup ; p.Ser698Arg/p.His158CysfsTer20 | 1 |
| 24 | NM_001139.2:c.467_470dup/c.1562A>G ; p.His158CysfsTer20/p.Tyr521Cys | 3 |
| 25 | NM_001139.2:c.467_470dup/c.467_470dup ; p.His158CysfsTer20/p.His158CysfsTer20 | 1 |
| 26 | NM_001139.2:c.808A>C/c.1562A>G ; p.Asn270His/p.Tyr521Cys | 1 |
|  | Ichthyosis, psoriasis, cardiomyopathies, atopic dermatitis |  |
| 27 | NM_001139.2:c.962T>A/c.1446dupC ; p.Met321Lys/p.Asn483GlnfsTer2 | 1 |
| 28 | NM_001139.2:c.962T>A/c.1562A>G ; p.Met321Lys/p.Tyr521Cys | 1 |
| 29 | NM_001139.2:c.962T>C/c.2094C>A ; p.Met321Thr/p.Ser698Arg | 1 |
| 30 | NM_001139.3:c.1156C>T/c.1790C>A ; p.Arg386Cys/p.Ala597Glu | 1 |
|  | Clinical data available in Pietrzak et al., 2022 |  |
| 31 | NM_001139.3:c.1562A>G/c.1163C>T ; p.Tyr521Cys/p.Ala388Val | 1 |
| 32 | NM_001139.3:c.1A>G/c.1790C>A ; p.Met1Val/p.Ala597Glu | 1 |
|  | ***ALOXE3*** | |
|  | Ichthyosis, congenital, autosomal recessive 3 (OMIM 606545), AR | |
| 1 | NM_021628.2:c.1076+1G>A/c.1103del ; p.?/p.? | 1 |
| 2 | NM_021628.2:c.1964del/c.1811A>G ; p.Leu655ArgfsTer93/p.Asn604Ser | 1 |
| 3 | NM_021628.2:c.38_41delACCT/c.700C>T ; p.Tyr13Ter/p.Arg234Ter | 1 |
| 4 | NM_021628.2:c.680+1G>A/c.842delG ; p.?/p.? | 1 |
| 5 | NM_021628.2:c.700C>T/c.1102-2A>T ; p.Arg234Ter/p.? | 1 |
| 6 | NM_021628.2:c.700C>T/c.1472T>G ; p.Arg234Ter/p.Leu491Arg | 1 |
| 7 | NM_021628.2:c.700C>T/c.1700T>C ; p.Arg234Ter/p.Leu567Pro | 1 |
| 8 | NM_021628.2:c.700C>T/c.1889C>T ; p.Arg234Ter/p.Pro630Leu | 1 |
| 9 | NM_021628.2:c.700C>T/c.680+1G>A ; p.Arg234Ter/p.? | 1 |
| 10 | NM_021628.2:c.700C>T/c.700C>T ; p.Arg234Ter/p.Arg234Ter | 7 |
| 11 | NM_021628.2:c.700C>T/c.952dup ; p.Arg234Ter/p.Leu318ProfsTer58 | 1 |
| 12 | NM_021628.2:c.700C>T/c.984C>A ; p.Arg234Ter/p.Tyr328Ter | 1 |
|  | ***CERS3*** | |
|  | Ichthyosis, congenital, autosomal recessive 9 (OMIM 615023), AR | |
| 1 | NM_001378789.1:c.(?_-1)_(410+1_411-1)del/c.(?_-1)_(410+1_411-1)del ; p.?/p.? | 1 |
|  | non-bullous ichthyotic erythroderma |  |
|  | ***CYP4F22*** | |
|  | Ichthyosis, congenital, autosomal recessive 5 (OMIM 604777 ), AR | |
| 1 | NM_173483:c.59dupG/c.59dupG ; p.(Ile21HisfsTer59)/p.(Ile21HisfsTer59) | 1 |
| 2 | NM_173483:c.667C>T/c.1190G>T ; p.Gln223Ter/p.Arg397Leu | 1 |
| 3 | NM_173483:c.667C>T/c.667C>T ; p.Gln223Ter/p.Gln223Ter | 1 |
|  | ***DSG1*** | |
|  | Erythroderma, congenital, with palmoplantar keratoderma, hypotrichosis, and hyper IgE (OMIM 615508), AR  Keratosis palmoplantaris striata I, AD (OMIM 148700), AD | |
| 1 | NM_001942.3:c.518-2A>G/- ; p.?/- | 1 |
|  | Clinical data available in Pietrzak et al., 2022 |  |
|  | ***DSP*** | |
|  | Arrhythmogenic right ventricular dysplasia 8 (OMIM 607450), AD  Cardiomyopathy, dilated, with woolly hair and keratoderma (OMIM 605676), AR  Dilated cardiomyopathy with woolly hair, keratoderma, and tooth agenesis (OMIM 615821), AD  Epidermolysis bullosa, lethal acantholytic (OMIM 609638), AR  Keratosis palmoplantaris striata II (OMIM 612908), AD | |
| 1 | NM_004415.4:c.7096C>T/c.7096C>T ; p.Arg2366Cys/p.Arg2366Cys | 1 |
|  | massive keratosis on the hands and feet, short, curly, fluffy hair |  |
|  | ***ERCC2*** | |
|  | Cerebrooculofacioskeletal syndrome 2 (OMIM 610756), AR  Trichothiodystrophy 1, photosensitive (OMIM 601675), AR  Xeroderma pigmentosum, group D (OMIM 278730), AR | |
| 1 | NM_00400.3:c.1703_1704delTT/c.2164C>T ; p.Phe568TyrfsTer2/p.Arg722Trp | 1 |
|  | ichthyosis, horizontal nystagmus, hypotrophy, delayed motor development |  |
|  | ***FLG*** | |
|  | Ichthyosis vulgaris (OMIM 146700), AD, AR  {Dermatitis, atopic, susceptibility to, 2} (OMIM 605803) | |
| 1 | NM_002016.1:c.1501C>T/c.1501C>T ; p.Arg501Ter/p.Arg501Ter | 1 |
| 2 | NM_002016.1:c.1501C>T/c.2282_2285delCAGT ; p.Arg501Ter/p.Ser761CysfsTer36 | 1 |
| 3 | NM_002016.1:c.1501C>T/c.7339C>T ; p.Arg501Ter/p.Arg2447Ter | 1 |
| 4 | NM_002016.1:c.2282_2285del/c.5690del ; p.Ser761CysfsTer36/p.His1897ProfsTer198 | 1 |
| 5 | NM_002016.1:c.2282_2285delCAGT/c.10255C>T ; p.Ser761CysfsTer36/p.Arg3419Ter | 1 |
| 6 | NM_002016.1:c.2282_2285delCAGT/c.1501C>T ; p.Ser761CysfsTer36/p.Arg501Ter | 2 |
| 7 | NM_002016.1:c.2282_2285delCAGT/c.2282_2285delCAGT ; p.Ser761CysfsTer36/p.Ser761CysfsTer36 | 2 |
| 8 | NM_002016.1:c.2282_2285delCAGT/c.6109C>T ; p.Ser761CysfsTer36/Arg2037Term | 1 |
|  | ***GJA1*** | |
|  | Craniometaphyseal dysplasia, autosomal recessive (OMIM 218400 ), AR  Erythrokeratodermia variabilis et progressiva 3 (OMIM 617525), AD  Oculodentodigital dysplasia (OMIM 164200), AD  Oculodentodigital dysplasia, autosomal recessive (OMIM 257850), AR  Palmoplantar keratoderma with congenital alopecia (OMIM 104100), AD  Syndactyly, type III (OMIM 186100), AD | |
| 1 | NM_000165.5:c.131C>T/- ; p.Ala44Val/- | 1 |
|  | ichthyosis hystrix |  |
|  | ***GJB2*** | |
|  | Bart-Pumphrey syndrome (OMIM 149200), AD  Deafness, autosomal dominant 3A (OMIM 601544), AD  Deafness, autosomal recessive 1A (OMIM 220290), AR, DD  Hystrix-like ichthyosis with deafness (OMIM 602540), AD  Keratitis-ichthyosis-deafness syndrome (OMIM 148210), AD  Keratoderma, palmoplantar, with deafness (OMIM 148350), AD  Vohwinkel syndrome (OMIM 124500), AD | |
| 1 | NM_004004.5:c.148G>A/- (mosaic); p.Asp50Asn/- | 1 |
| 2 | NM_004004.5:c.35del/c.161A>G ; p.Gly12ValfsTer2/p.Asn54Ser | 1 |
|  | PPK, hearing loss |  |
|  | ***GJB3*** | |
|  | Deafness, autosomal dominant 2B, with or without peripheral neuropathy (OMIM 612644), AD  Deafness, digenic, GJB2/GJB3 (OMIM 220290), AR, DD  Erythrokeratodermia variabilis et progressiva 1 (OMIM 133200), AD, AR | |
| 1 | NM_024009.3:c.125G>C/- ; p.Arg42Pro/- | 1 |
|  | PPK, generalised erythrodermia |  |
|  | ***GJB4*** | |
|  | Erythrokeratodermia variabilis et progressiva 2 (OMIM 617524), AD | |
| 1 | NM_153212.2:c.35G>A/- ; p.Gly12Asp/- | 1 |
|  | familial keratoderma |  |
|  | ***KRT1*** | |
|  | Epidermolytic hyperkeratosis 1 (OMIM 113800), AD  Ichthyosis histrix, Curth-Macklin type (OMIM 146590), AD  Ichthyosis, annular epidermolytic 2 (OMIM 620148), AD  Keratosis palmoplantaris striata III (OMIM 607654 ),  Palmoplantar keratoderma, epidermolytic, 2 (OMIM 620411), AD  Palmoplantar keratoderma, nonepidermolytic (OMIM 600962), AD | |
| 1 | NM_006121.4:c.1430T>C/- ; p.Leu477Pro/- | 1 |
|  | familial keratinisation disorder |  |
| 2 | NM_006121.4:c.1436T>C/- ; p.Ile479Thr/- | 1 |
|  | PPK |  |
| 3 | NM_006121.4:c.1535delT/- ; p.Ile512ThrfsTer102/- | 1 |
|  | Clinical data available in Pietrzak et al., 2022 |  |
| 4 | NM_006121.4:c.539A>T/- ; p.Glu180Va/- | 1 |
|  | keratoderma |  |
| 5 | NM_006121.4:c.551T>A/- ; p.Ile184Asn/- | 1 |
|  | bullous ichthyosis |  |
| 6 | NM_006121.4:c.591+1G>A/- ; p.?/- | 1 |
|  | bullous ichthyosis (Clinical data available in Osipowicz et al., 2020) |  |
|  | ***KRT10*** | |
|  | Ichthyosis histrix, Lambert type (?) (OMIM 146600), AD  Epidermolytic hyperkeratosis 2A, autosomal dominant (OMIM 620150), AD  Epidermolytic hyperkeratosis 2B, autosomal recessive (OMIM 620707), AR  Ichthyosis with confetti (OMIM 609165), AD  Ichthyosis, annular epidermolytic 1 (OMIM 607602), AD | |
| 1 | NM_000421.3:c.1554dupC/- ; p.Ser519GlnfsTer62/- | 1 |
|  | Ichthyosis with confetti |  |
| 2 | NM_000421.3:c.1689_1690del/- ; p.Ser563ArgfsX17/- | 1 |
|  | bullous ichthyosis |  |
| 3 | NM_000421.3:c.449T>C/- ; p.Met150Thr/- | 1 |
|  | Ichthyosis |  |
| 4 | NM_000421.3:c.457C>G/- ; p.Leu153Val/- | 1 |
|  | familial ichthyosis |  |
| 5 | NM_000421.3:c.466C>T/- ; p.Arg156Cys/- | 3 |
|  | - ichthyosis (2 patients) |  |
|  | - separation of the epidermis with flakes from the first day of life (1 patient) |  |
| 6 | NM_000421.3:c.467G>A/- ; p.Arg156His/- | 7 |
|  | - mosaic - linear keratoderma (1 patient) |  |
|  | - bullous ichthyosis (3 patients) |  |
|  | - thickening of the epidermis on the elbows and knees, separation of the epidermis all over the body with erythroderma of the entire skin, with temporary improvement, periodically intensified exfoliation of the epidermis in places of pressure/rubbing (1 patient) |  |
|  | - bullous ichthyosis (Clinical data available in Osipowicz et al., 2020) (1 patient) |  |
|  | - ichthyosis, other atopic dermatitis, psoriasis (1 patient) |  |
| 7 | NM_000421.5: c.1441_1448del/- ; p.Gly481ArgfsTer97/- | 1 |
|  | ***KRT16*** | |
|  | Pachyonychia congenita 1 (OMIM 167200), AD 3 Palmoplantar keratoderma, nonepidermolytic, focal (OMIM 613000), AD | |
| 1 | NM_005557.3:c.373A>G/- ; p.Asn125Asp/- | 1 |
|  | palmar keratoderma |  |
|  | ***KRT2*** | |
|  | Ichthyosis bullosa of Siemens (OMIM 146800 AD | |
| 1 | NM_000423.2:c.1459G>A/- ; p.Glu487Lys/- | 1 |
|  | ichthyosis |  |
| 2 | NM_000423.2:c.1912T>C/- ; p.Phe638Leu/- | 1 |
|  | familial ichthiosis |  |
| 3 | NM_000423.2:c.566T>C/- ; p.Phe189Ser/- | 1 |
|  | ichthyosis, features of erythroderma |  |
|  | ***KRT9*** | |
|  | Palmoplantar keratoderma, epidermolytic, 1 (OMIM 144200), AD | |
| 1 | NM_000226.3:c.482A>G/- ; p.Asn161Ser/- | 3 |
| 2 | NM_000226.3:c.487C>T/- ; p.Arg163Trp/- | 1 |
| 3 | NM_000226.3:c.488G>A/- ; p.Arg163Gln/- | 10 |
|  | - Clinical data available in Pietrzak et al., 2022 (1 patient) |  |
|  | - PPK (1 patient) |  |
|  | - PPK, blisters (1 patient) |  |
|  | ***LORICRIN*** | |
|  | Vohwinkel syndrome with ichthyosis (OMIM 604117), AD | |
| 1 | NM_000427.2:c.639_642dup/- ; p.Thr215GlyfsTer122/- | 2 |
|  | Clinical data available in Wertheim-Tysarowska et al., 2023 (1 patient) |  |
|  | congenital ichthyosis (1 patient) |  |
|  | ***NIPAL4*** | |
|  | Ichthyosis, congenital, autosomal recessive 6 (OMIM 612281), AR | |
| 1 | NM_001099287.2:c.341C>A/c.341C>A ; p.Ala114Asp/p.Ala114Asp | 3 |
| 2 | NM_001099287.2:c.341C>A/c.463+5G>A ; p.Ala114Asp/p.? | 1 |
| 3 | NM_001099287.2:c.341C>A/c.658G>A ; p.Ala114Asp/p.Gly220Arg | 1 |
|  | ***PNPLA1*** | |
|  | Ichthyosis, congenital, autosomal recessive 10 (OMIM 615024), AR | |
| 1 | NM_001145717.1:c.275delC/c.275delC ; p.Pro92ArgfsTer8/p.Pro92ArgfsTer8 | 1 |
| 2 | NM_001145717.1:c.301A>G/c.301A>G ; p.Arg101Gly/p.Arg101Gly | 1 |
|  | ***SLC27A4*** | |
|  | Ichthyosis prematurity syndrome (OMIM 608649), AR | |
| 1 | NM_005094.3:c.1541A>G/c.1510C>T ; p.Glu514Gly/p.Arg504Cys | 1 |
| 2 | NM_005094.3:c.931C>T/c.931C>T ; p.Arg311Trp/p.Arg311Trp | 1 |
|  | ichthyosis, mild intelectual disability |  |
|  | ***SPINK5*** | |
|  | Netherton syndrome (OMIM 256500), AR | |
| 1 | NM_006846.3:c.1431-12G>A/c.1530C>A ; p.?/p.Cys510Ter | 1 |
| 2 | NM_006846.3:c.1530C>A/c.(?_-1)_(410+1_411-1)del  ; p.Cys510Ter/p.? | 1 |
| 3 | NM_006846.3:c.1816_1820+21delinsCT/c.(?_-1)_(1479+1_1480-1)del ; p.?/p.? | 1 |
| 4 | NM_006846.3:c.1825C>T/c.(?_-1)_(1479+1_1480-1)del ; p.Gln609Ter/p.? | 1 |
| 5 | NM_006846.3:c.2098G>T/- ; p.Gly700Ter/c.2468delA | 1 |
| 6 | NM_006846.3:c.2098G>T/c.(?_-1)_(1479+1_1480-1)del ; p.Gly700Ter/p.? | 1 |
| 7 | NM_006846.3:c.410+1G>A/c.410+1G>A ; p.?/p.? | 1 |
| 8 | NM_006846.3:c.649C>T/c.411-5T>A ; p.Arg217Ter/p.? | 1 |
| 9 | NM_006846.3:c.715dupT/c.1431-12G>A ; p.Cys239LeufsTer6/p.? | 1 |
| 10 | NM_021628.2: c.(?_-1)_(410+1_411-1)del/ c.(?_-1)_(410+1_411-1)del  ; p.?/p.? | 1 |
|  | ***STS*** | |
|  | Ichthyosis, X-linked (OMIM 308100), XLR | |
| 1 | NM_000351.4:c.(?_-1)_(*1_?)del ; p.1Met_583Terdel | 26 |
|  | ***TGM1*** | |
|  | Ichthyosis, congenital, autosomal recessive 1 (OMIM 242300 ), AR | |
| 1 | NM_000359.2:c.1135G>C/c.(1402+1_1401-1)_(2225+1_2226-1)dup ; p.Val379Leu/p.? | 1 |
| 2 | NM_000359.2:c.1135G>C/c.1135G>C ; p.Val379Leu/p.Val379Leu | 1 |
| 3 | NM_000359.2:c.1135G>C/c.1186C>T ; p.Val379Leu/p.Arg396Cys | 1 |
| 4 | NM_000359.2:c.1135G>C/c.1490A>T ; p.Val379Leu/p.Glu497Val | 1 |
| 5 | NM_000359.2:c.1135G>C/c.1500T>A ; p.Val379Leu/p.Ser500Arg | 1 |
| 6 | NM_000359.2:c.1135G>C/c.2059C>T ; p.Val379Leu/p.Arg687Cys | 1 |
| 7 | NM_000359.2:c.1166G>C/c.377G>A ; p.Arg389Pro/p.Arg126His | 1 |
| 8 | NM_000359.2:c.1500T>A/c.(1402+1_1401-1)_(2225+1_2226-1)dup ; p.Ser500Arg/p.? | 1 |
| 9 | NM_000359.2:c.2059C>T/c.(1402+1_1401-1)_(2225+1_2226-1)dup ; p.Arg687Cys/p.? | 1 |
| 10 | NM_000359.2:c.316C>T/c.1166G>C ; p.Arg106Ter/p.Arg389Pro | 1 |
| 11 | NM_000359.2:c.376C>T/c.1187G>T ; p.Arg126Cys/p.Arg396Leu | 1 |
| 12 | NM_000359.2:c.377G>A/c.(1402+1_1401-1)_(2225+1_2226-1)dup ; p.Arg126His/p.? | 4 |
| 13 | NM_000359.2:c.377G>A/c.1135G>C ; p.Arg126His/- | 1 |
| 14 | NM_000359.2:c.377G>A/c.1135G>C ; p.Arg126His/p.Val379Leu | 2 |
| 15 | NM_000359.2:c.377G>A/c.377G>A ; p.Arg126His/p.Arg126His | 4 |
| 16 | NM_000359.2:c.377G>A/c.877-2A>G ; p.Arg126His/p.? | 1 |
| 17 | NM_000359.2:c.424C>T/c.877-2A>G ; p.Arg142Cys/p.? | 1 |
| 18 | NM_000359.2:c.428G>T/c.1159+1G>A ; p.Arg143Leu/p.? | 1 |
| 19 | NM_000359.2:c.579G>A/c.1135G>C ; p.Trp193Ter/p.Val379Leu | 1 |
| 20 | NM_000359.2:c.579G>A/c.579G>A ; p.Trp193Ter/p.Trp193Ter | 1 |
| 21 | NM_000359.2:c.579G>A/c.877-2A>G ; p.Trp193Ter/p.? | 1 |
| 22 | NM_000359.2:c.788G>A/c.1135G>C ; p.Trp263Ter/p.Val379Leu | 1 |
|  | Clinical data available in Pietrzak et al., 2022 |  |
| 23 | NM_000359.2:c.876+2T>C/c.877-2A>G ; p.?/p.? | 1 |
| 24 | NM_000359.2:c.919C>T/c.(1402+1_1401-1)_(2225+1_2226-1)dup ; p.Arg307Trp/p.? | 1 |
| 25 | NM_000359.2:c.943C>T/c.(1402+1_1401-1)_(2225+1_2226-1)dup ; p.Arg315Cys/p.? | 1 |
| 26 | NM_000359.2:c.968G>A/c.425G>A ; p.Arg323Gln/p.Arg142His | 1 |
|  | ***POMP*** | |
|  | Keratosis linearis with ichthyosis congenita and sclerosing keratoderma (OMIM 601952), AR  Proteasome-associated autoinflammatory syndrome 2 (OMIM 618048), AD | |
| 1 | NM_015932.6:c.-95delC/c.-95delC ; p.?/p.? | 1 |
|  | generalized exfoliation, eyes - core fibers, myopia, astigmatism, itching |  |

AR – autosomal recessive inheritance , AD – autosomal Dominant inheritance, XLR – X-linked recessive inheritance, OMIM refers to OMIM database number (<https://www.omim.org/>) , p.? - indicates that an effect on the protein level is expected, but that it is not possible to give a reliable prediction of the consequences,

References:

Pietrzak A, Wawrzycki B, Schmuth M, Wertheim-Tysarowska K. Structural and functional foot disorders in patients with genodermatoses: a single-centre, retrospective chart review. Orphanet J Rare Dis. 2022;17:53.

Osipowicz K, Wertheim-Tysarowska K, Kwiek B, Jankowska E, Gos M, Charzewska A, Woźniak K, Kowalewski C. Bullous diseases caused by *KRT1* gene mutations: from epidermolytic hyperkeratosis to a novel variant of epidermolysis bullosa simplex. Postepy Dermatol Alergol. 2021;38:1032-1038.

Wertheim-Tysarowska K, Osipowicz K, Gielniewski B, Wojtaś B, Szabelska-Beręsewicz A, Zyprych-Walczak J, Mika A, Tysarowski A, Duk K, Rygiel AM, Niepokój K, Woźniak K, Kowalewski C, Wierzba J, Jezela-Stanek A. The Epidermal Transcriptome Analysis of a Novel c.639_642dup *LORICRIN* Variant-Delineation of the Loricrin Keratoderma Pathology. Int J Mol Sci. 2023;24:9459.
