# Supplementary material for: Molecular analysis of inherited disorders of cornification in polish patients show novel variants and functional data and provokes questions on the significance of secondary findings
Source: Orphanet J Rare Dis. 2024 Nov 5;19:413. doi: 10.1186/s13023-024-03395-4 (PMC11536877; doi:10.1186/s13023-024-03395-4)
Supplement: Supplementary file 4 — Supplementary Material 4 [file 13023_2024_3395_MOESM4_ESM.docx]

Additional File 4.

The list of recurrent variants detected in the study.

| **Gene** | **Variant name** | **Number of alleles** |
| --- | --- | --- |
| *ALOX12B* | c.1562A>G (p.Tyr521Cys) | 53 (1 as SF) |
| *STS* | c.(?_-1)_(*1_?)del (p.1Met_583Terdel) | 26 |
| *ALOXE3* | c.700C>T (p.Arg234Ter) | 22 |
| *FLG* | c.2282_2285delCAGT (p.Ser761CysfsTer36) | 19 (6 as SF) |
| *TGM1* | c.377G>A (p.Arg126His) | 18 (1 as SF) |
| *TGM1* | c.1135G>C (p.Val379Leu) | 12 |
| *ALOX12B* | c.1790C>A (p.Ala597Glu) | 11 |
| *FLG* | c.1501C>T (p.Arg501Ter) | 11 |
| *KRT9* | c.488G>A (p.Arg163Gln) | 10 |
| *ALOX12B* | c.1A>G (p.Met1Val) | 9 |
| *NIPAL4* | c.341C>A (p.Ala114Asp) | 9 (1 as SF) |
| *TGM1* | c.(1402+1_1401-1)_(2225+1_2226-1)dup (p.?) | 9 |
| *ALOX12B* | c.2094C>A (p.Ser698Arg) | 7 |
| *ALOX12B* | c.467_470dup (p.His158CysfsTer20) | 7 |
| *KRT10* | c.467G>A (p.Arg156His) | 7 |
| *ALOX12B* | c.1265C>T (p.Pro422Leu) | 6 |
| *TGM1* | c.579G>A (p.Trp193Ter) | 4 |
| *TGM1* | c.877-2A>G (p.?) | 4 |
| *ABCA12* | c.4543C>T (p.Arg1515Ter) | 3 |
| *ALOX12B* | c.1163C>T (p.Ala388Val) | 4 |
| *CYP4F22* | c.667C>T (p.Gln223Ter) | 3 |
| *FLG* | c.7339C>T (p.Arg2447Ter) | 3 (2 as SF) |
| *KRT10* | c.466C>T (p.Arg156Cys) | 3 |
| *KRT9* | c.482A>G (p.Asn161Ser) | 3 |
| *SPINK5* | ex1-16del | 3 |
| *SPINK5* | ex1-5del | 3 |
| *ABCA12* | c.2140C>T (p.Arg714Ter) | 2 |
| *ABCA12* | c.4139A>G (p.Asn1380Ser) | 2 |
| *ABCA12* | c.179G>C (p.Arg60Pro) | 2 (1 as SF) |
| *ABCA12* | c.6611G>A (p.Arg2204Gln) | 2 (1 as SF) |
| *ALDH3A2* | c.734A>C (p.Asp245Ala) | 2 |
| *ALOX12B* | c.1579G>A (p.Val527Met) | 2 |
| *ALOX12B* | c.1454T>C (p.Phe485Ser) | 2 |
| *ALOX12B* | c.962T>A (p.Met321Lys) | 2 |
| *ALOXE3* | c.680+1G>A (p.?) | 2 |
| *CERS3* | ex12del | 2 |
| *CYP4F22* | c.59dupG (p.(Ile21HisfsTer59) | 2 |
| *DSP* | c.7096C>T (p.Arg2366Cys) | 2 |
| *FLG* | c.6109C>T (p.Arg2037Ter) | 2 |
| *LORICRIN* | c.639_642dup (p.Thr215GlyfsTer122) | 2 |
| *PNPLA1* | c.275delC (p.Pro92ArgfsTer8) | 2 |
| *PNPLA1* | c.301A>G (p.Arg101Gly) | 2 |
| *POMP* | c.-95delC (p.?) | 2 |
| *SLC27A4* | c.931C>T (p.Arg311Trp) | 2 |
| *SPINK5* | c.2098G>T (p.Gly700Ter) | 2 |
| *SPINK5* | c.1431-12G>A (p.?) | 2 |
| *SPINK5* | c.1530C>A (p.Cys510Ter) | 2 |
| *SPINK5* | c.410+1G>A (p.?) | 2 |
| *TGM1* | c.2059C>T (p.Arg687Cys) | 2 |
| *TGM1* | c.1166G>C (p.Arg389Pro) | 2 |
| *TGM1* | c.1500T>A (p.Ser500Arg) | 2 |
