# Supplementary material for: Molecular analysis of inherited disorders of cornification in polish patients show novel variants and functional data and provokes questions on the significance of secondary findings
Source: Orphanet J Rare Dis. 2024 Nov 5;19:413. doi: 10.1186/s13023-024-03395-4 (PMC11536877; doi:10.1186/s13023-024-03395-4)
Supplement: Supplementary file 6 — Supplementary Material 6 [file 13023_2024_3395_MOESM6_ESM.docx]

Additional file 6

The pathways over-represented in transcriptomic analysis of MeDOC patients

| **Pathway name** | **Entities found** | **EntitiesTotal** | **Entities ratio** | **Entities pValue** | **Entities FDR** | **Reactions found** | **Reactions total** | **Reactions ratio** |
| --- | --- | --- | --- | --- | --- | --- | --- | --- |
| Keratinization | 55 | 226 | 0.015 | 1.11E-16 | 5.46E-14 | *29* | *34* | *0.002* |
| Formation of the cornified envelope | 54 | 138 | 0.009 | 1.11E-16 | 5.46E-14 | *22* | *27* | *0.002* |
| Interleukin-36 pathway | 5 | 7 | 0 | 2,00E-05 | 6.23E-3 | *3* | *3* | *0* |
| Metal sequestration by antimicrobial proteins | 5 | 13 | 0.001 | 3.37E-4 | 8.29E-2 | *4* | *5* | *0* |
| TP53 Regulates Transcription of Genes Involved in G2 Cell Cycle Arrest | 5 | 21 | 0.001 | 2.77E-3 | 4.56E-1 | *6* | *11* | *0.001* |
| Gap junction assembly | 7 | 41 | 0.003 | 2.78E-3 | 4.56E-1 | *8* | *16* | *0.001* |
| Polo-like kinase mediated events | 5 | 23 | 0.001 | 4.07E-3 | 5.69E-1 | *11* | *15* | *0.001* |
| Phosphorylation of Emi1 | 3 | 8 | 0.001 | 5.82E-3 | 6.97E-1 | *2* | *2* | *0* |
| Post-translational modification: synthesis of GPI-anchored proteins | 12 | 115 | 0.007 | 6.49E-3 | 6.97E-1 | *1* | *16* | *0.001* |
| Interferon gamma signaling | 16 | 177 | 0.011 | 7.11E-3 | 6.97E-1 | *2* | *23* | *0.002* |
| Gap junction trafficking | 7 | 52 | 0.003 | 9.73E-3 | 7.67E-1 | *8* | *20* | *0.001* |
| G1/S-Specific Transcription | 6 | 43 | 0.003 | 1.38E-2 | 7.67E-1 | *6* | *28* | *0.002* |
| Regulation of TP53 Expression | 2 | 4 | 0 | 1.42E-2 | 7.67E-1 | *4* | *5* | *0* |
| Interferon alpha/beta signaling | 12 | 129 | 0.008 | 1.49E-2 | 7.67E-1 | *2* | *25* | *0.002* |
| Gap junction trafficking and regulation | 7 | 57 | 0.004 | 1.54E-2 | 7.67E-1 | *8* | *24* | *0.002* |
| Neutrophil degranulation | 32 | 478 | 0.031 | 1.71E-2 | 7.67E-1 | *9* | *10* | *0.001* |
| Antimicrobial peptides | 11 | 123 | 0.008 | 2.46E-2 | 7.67E-1 | *17* | *58* | *0.004* |
| Regulation of HMOX1 expression and activity | 2 | 6 | 0 | 3.01E-2 | 7.67E-1 | *4* | *4* | *0* |
| OAS antiviral response | 3 | 16 | 0.001 | 3.59E-2 | 7.67E-1 | *6* | *15* | *0.001* |
| FCGR activation | 9 | 103 | 0.007 | 4.46E-2 | 7.67E-1 | *6* | *6* | *0* |
