# Supplementary figures and images for: Molecular analysis of inherited disorders of cornification in polish patients show novel variants and functional data and provokes questions on the significance of secondary findings
Source: Orphanet J Rare Dis. 2024 Nov 5;19:413. doi: 10.1186/s13023-024-03395-4 (PMC11536877; doi:10.1186/s13023-024-03395-4)

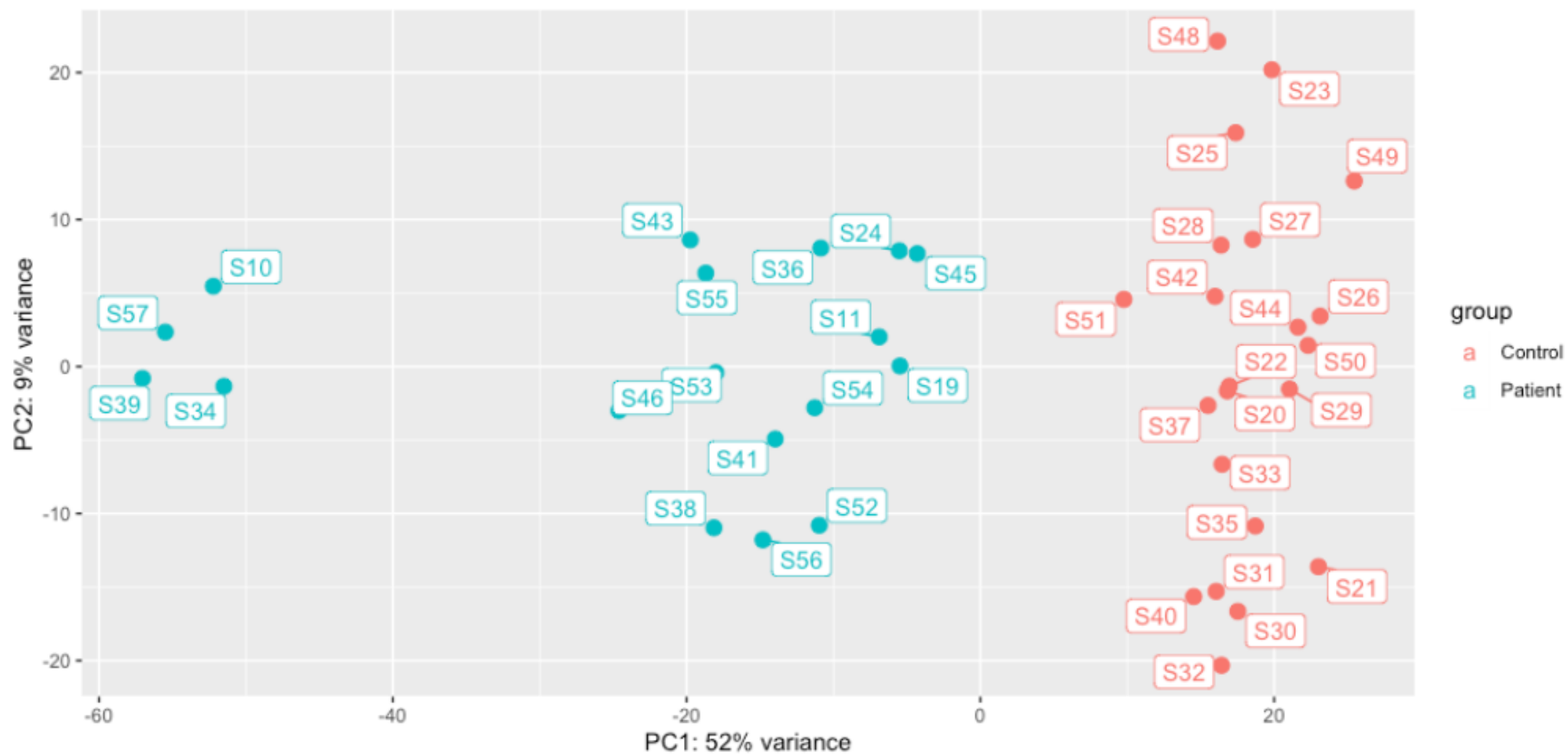

Supplement: Supplementary file 7 — Supplementary Material 7 [file 13023_2024_3395_MOESM7_ESM.pdf]
